# Hcfc1 and Ogt Mediate Zebrafish CNS Regeneration Through Hippo/Yap Signalling

**DOI:** 10.1111/cpr.70132

**Published:** 2025-10-17

**Authors:** Priyanka P. Srivastava, Sidharth Bhasin, Poonam Sharma, Omkar Mahadeo Desai, Kshitiz Yadav, Ayushma, Rohan Chakraborty, Suhel Parvez, Rajesh Ramachandran, Shilpi Minocha

**Affiliations:** ^1^ Kusuma School of Biological Sciences, Indian Institute of Technology Delhi (IITD) New Delhi India; ^2^ Center for Regenerative Therapies Dresden (CRTD), Technische Universität Dresden Dresden Germany; ^3^ Department of Biological Sciences Indian Institute of Science Education and Research Mohali India; ^4^ Model Systems for Infection and Immunity (MSYS) Helmholtz Centre for Infection Research Braunschweig Germany; ^5^ Department of Medical Elementology and Toxicology Jamia Hamdard University New Delhi India

**Keywords:** brain, Hcfc1, Hippo/Yap signalling, Ogt, regeneration, retina, zebrafish

## Abstract

Regeneration of the central nervous system (CNS) is a complex and tightly regulated process, yet the precise molecular players and transcriptional regulators involved remain incompletely understood. Here, we identify Host Cell Factor‐1 (Hcfc1), a transcriptional co‐regulator, and *O*‐GlcNAc transferase (Ogt), which cleaves and *O*‐GlcNAcylates HCF‐1, as crucial regulators of zebrafish brain and retinal regeneration. We uncover their interplay with the Hippo/Yap signalling pathway, a well‐known regulator of tissue growth and repair. Knockdown of *hcfc1a/b* or Ogt activity inhibition disrupts regeneration and reduces Yap levels, while Yap inhibition alone also impairs regeneration. Strikingly, overexpression of constitutively active Yap5SA rescues proliferation defects caused by Hcfc1 depletion and Ogt inhibition in retinal regeneration. Further, *yap1* knockdown reduces *hcfc1a/b* levels, suggesting potential feedback regulation. These findings reveal a previously unrecognised regulatory axis involving Hcfc1, Ogt, and the Hippo/Yap pathway, which governs CNS regeneration. Targeting this pathway could offer a strategy for enhancing CNS regeneration.

## Graphical abstract

This work identifies Hcfc1 and Ogt as key regulators of zebrafish CNS regeneration through modulation of Hippo/Yap signalling. Loss of Hcfc1 or Ogt activity inhibition impairs regeneration, which is restored by Yap overexpression, revealing a new regulatory axis that enhances CNS regenerative capacity.

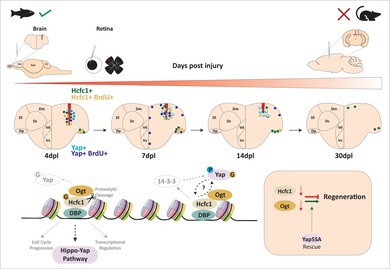

## 1 Introduction

Injuries to the central nervous system (CNS), including the brain and retina, often result in irreversible cellular and functional loss, with available treatments primarily focusing on symptom management and pain relief. Over the course of evolution, mammals have lost much of their regenerative potential [1, 2, 3, 4, 5, 6, 7], with limited availability of neurogenic niches identified in the hippocampus and subventricular zone (SVZ) [8, 9]. Also, the mammalian retina is largely considered to lack neurogenic capacity. However, recent studies have identified a population of undifferentiated cells with stem cell characteristics in the ciliary body epithelium of adult rats and other mammals [10, 11], and similar cells have been observed in adult monkeys and humans, exhibiting molecular markers of retinal stem/progenitors [12, 13].

Teleost fish, such as zebrafish, possess remarkable CNS regenerative potential due to the presence of constitutively active neurogenic niches throughout the CNS. Extensive research has been dedicated to understanding the molecular and cellular mechanisms underlying CNS regeneration in these species, though many aspects remain poorly understood [14, 15, 16, 17, 18, 19, 20, 21, 22, 23, 24, 25, 26]. Zebrafish share over 70% genetic similarity with humans, including orthologs of key genes involved in neural development and regeneration. Studies have demonstrated that zebrafish can regenerate both brain and retinal tissues through the proliferation and differentiation of neural precursor cells (NPCs), making them a valuable model for exploring regenerative mechanisms that may be applicable to non‐regenerative species [27, 28, 29, 30, 31, 32, 33].

Differentiation and proliferation of neural precursor cells (NPC) are key processes involved in the initiation of CNS regeneration. Recent studies have implicated the role of a unique and conserved transcriptional coregulator, Host cell factor 1 (HCF‐1), in controlling NPC proliferation and differentiation [34, 35, 36]. Initially discovered as an accessory protein for the VP16 transcription factor of herpes simplex virus (HSV) [37], HCF‐1 is now recognised as a key cellular coactivator with wide‐ranging effects on transcription and cell cycle regulation [38, 39, 40]. HCF‐1 protein is a 2035 amino acid‐long precursor containing a Kelch domain, six HCF‐1‐PRO repeats, and a fibronectin‐like domain, with proteolytic cleavage being essential for its function [41]. The enzyme OGT (*O*‐Linked *N*‐Acetylglucosamine (GlcNAc) Transferase) plays a crucial role in HCF‐1 cleavage, generating two subunits: HCF‐1n, which is involved in G1 phase progression, and HCF‐1c, which is essential for mitotic exit [42, 43, 44, 45]. Interestingly, HCF‐1 is known to have diverse target genes and binds to 5400 promoters, which suggests its role as a “master transcriptional regulator” [46]. Mutations in the human *HCFC1* gene cause methylmalonic acidemia and homocysteinemia, cblX type (cblX), an X‐linked recessive disorder characterised by defects in cobalamin (vitamin B12) metabolism, neurological impairment, intractable epilepsy, and failure to thrive [35, 36, 47, 48]. Deletion of HCF‐1 in Nkx2.1‐positive progenitors in the murine ventral telencephalon leads to cortical defects resembling unilateral polymicrogyria, reduced GABAergic interneurons, and glial cell loss. These defects largely arise due to increased apoptosis before cell migration, highlighting the essential role of HCF‐1 in murine brain development [49].

Zebrafish possess two orthologs of human HCFC1 (mouse HCF‐1), namely Hcfc1a and Hcfc1b, which exhibit substantial homology to human HCFC1. Both genes have been implicated in NPC and neural crest cell (NCC) proliferation and differentiation during development [50, 51, 52]. During regeneration, the activation of developmental processes may diverge from their functions during development or engage specific injury‐related pathways. This has prompted our investigation into the roles of Hcfc1 and its interacting partner, Ogt, in CNS regeneration, specifically within the brain and retina.

This study investigates the functional roles of Hcfc1 and Ogt in zebrafish brain and retinal regeneration. We show that knockdown of zebrafish *hcfc1a* and *hcfc1b*, as well as pharmacological inhibition of Ogt, leads to impaired regenerative outcomes, which are associated with disruptions in the Hippo/Yap signalling pathway. Furthermore, we explore how modulation of Yap activity can rescue regenerative defects as a potential mediator of Hcfc1's regulatory functions. Our findings provide new insights into the molecular mechanisms governing CNS regeneration and highlight Hcfc1 and Ogt as critical regulators of this process.

## 2 Results

### 2.1 Injury‐Induced Brain Regeneration Model in Zebrafish

The telencephalic stab wound injury model was employed to study brain regeneration in zebrafish, as previously described [17, 18, 19, 28, 53]. This model involves inducing a localised stab wound lesion in the dorsal part of the telencephalic ventricular zone and the parenchyma region, which is typically devoid of newborn neurons and glial progenitors under physiological conditions, and hence allows for the assessment of the regenerative response following injury. The contralateral left hemisphere serves as an internal control to evaluate the extent of regenerative activity [18]. The poke site could be visibly differentiated upon TBO staining, reflecting the accumulation of cells upon lesion (Figure S1). Zebrafish were sacrificed at multiple time points (1, 4, 7, 14, and 30 days post‐lesion, dpl). The 1 dpl samples represent the acute phase, characterised by significant tissue disruption, reorganisation of cellular populations, vacuolization near the lesion, and cerebral edema. At 4 and 7 dpl, cells around the lesion site proliferate in response to injury, marking the early stages of regeneration. Further, the injury site was visibly less prominent by 30 dpl (Figure S1). To examine the cellular response to injury, we analysed the expression of several key markers. Proliferation was assessed using proliferating cell nuclear antigen (PCNA) and bromodeoxyuridine (BrdU). We observed elevated PCNA expression at 4 and 7 dpl, with peak expression at 7 dpl, which then decreased at 14 dpl (Figure S1). Additionally, BrdU incorporation was significantly higher in the lesioned hemisphere compared to the contralateral unlesioned side. This increase in BrdU‐positive cells in the lesioned telencephalic hemisphere further confirms injury‐induced proliferation (Figure S1). Using this model, we now aim to explore the underlying molecular and cellular mechanisms driving the process of regeneration.

### 2.2 Hcfc1 and Ogt Is Upregulated During Zebrafish Brain Regeneration

Following the establishment of the telencephalic stab‐wound injury model, we sought to explore the molecular mechanisms driving brain regeneration, particularly focusing on Hcfc1. HCF‐1 has been implicated in cellular processes like proliferation and differentiation [35, 36], which are critical during tissue regeneration. Given its potential role in these processes, we decided to investigate its expression and function during brain regeneration in zebrafish. Initially, we examined the mRNA expression of both *hcfc1a* and *hcfc1b* paralogs in the adult zebrafish brain and found that *hcfc1b* is predominantly expressed compared to *hcfc1a* (Figure S2A). Immunostaining with an Hcfc1‐specific antibody [51, 54], predicted to recognize both Hcfc1a and Hcfc1b, revealed widespread Hcfc1 expression throughout the adult zebrafish telencephalon (Figure S2B).

**FIGURE 1:**
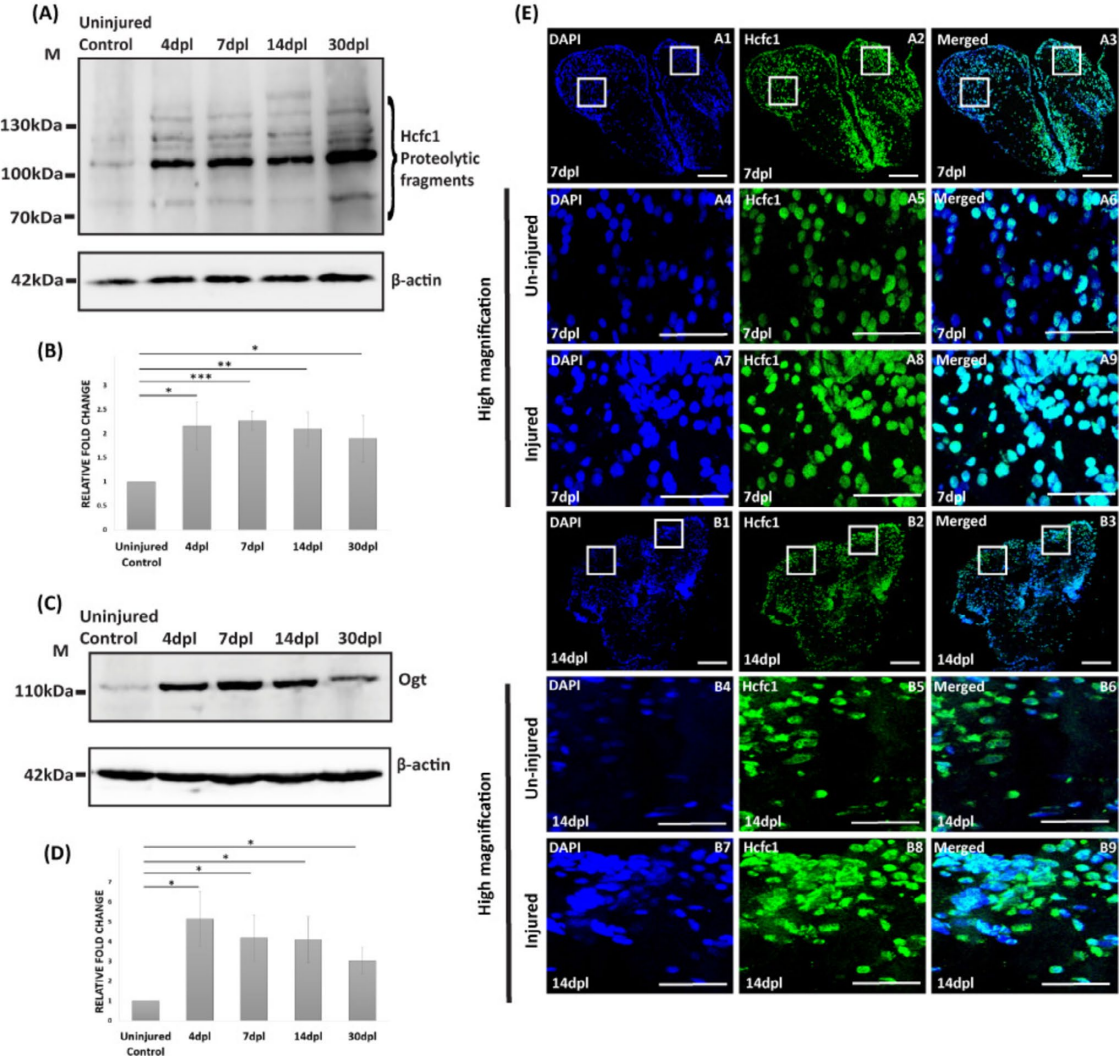
Hcfc1 And Ogt are upregulated upon injury‐induced brain regeneration: (A) Immunoblot of regenerating brains (uninjured control, 4, 7, 14 and 30 dpl) with anti‐HCFC1. (B) Quantification of Hcfc1 levels during regeneration is shown as a bar graph (*n* = 4), (C) Immunoblot of regenerating brains (uninjured control, 4, 7, 14 and 30 dpl) with anti‐OGT, (D) Quantification of Ogt levels during regeneration is shown as a bar graph (*n* = 3). Anti‐β‐Actin was used as a loading control. Significance is represented as * for *p*‐value < 0.05, ** for *p*‐value < 0.01 and *** for *p*‐value < 0.001 for all the quantifications. (E) Immunostaining of regenerating brains at 7 dpl (Panel A) and 14 dpl (Panel B) with anti‐HCFC1(green) and counterstained with DAPI (blue). Scale bar for A1–A3 and B1–B3 is 100 μm and scale bar for A4–A9 and B4–B9 50 μm.

Next, we examined the temporal expression of Hcfc1 during brain regeneration. Immunoblotting revealed a significant upregulation of Hcfc1 upon regeneration at 4 days post‐lesion (dpl) and 7 dpl, with its protein levels declining by 14 dpl (Figure 1A,B). This prompted us to investigate levels of Ogt (*O*‐linked *N*‐acetylglucosamine transferase), a key enzyme that is known to activate Hcfc1 through proteolytic cleavage. We observed an increase in Ogt expression starting at 4 dpl, and the increase in expression was consistent with that of Hcfc1. Notably, the majority of the upregulated Ogt expression co‐localised spatiotemporally with that of Hcfc1(Figure 1C,D, and Figure S3A). Interestingly, we also measured *O*‐GlcNAcylation levels using the RL2 antibody, which showed a modest upregulation, indicating that *O*‐GlcNAcylation may be specifically involved in the regulation of proteins crucial for regeneration (Figure S3B,C).

To investigate the spatial expression of Hcfc1, we performed immunostaining on brain sections and observed an increased accumulation of Hcfc1‐positive cells at the injury site in regenerating brains with predominant nuclear localization (Figure 1E). Immunostaining results were consistent with immunoblot data, showing higher Hcfc1 expression at 7 dpl compared to 14 dpl, but still significantly elevated in the injured hemisphere relative to the contralateral control hemisphere (Figure 1E panel A8 vs. B8 and Figure 1E panel B5 vs. B8).

**FIGURE 2:**
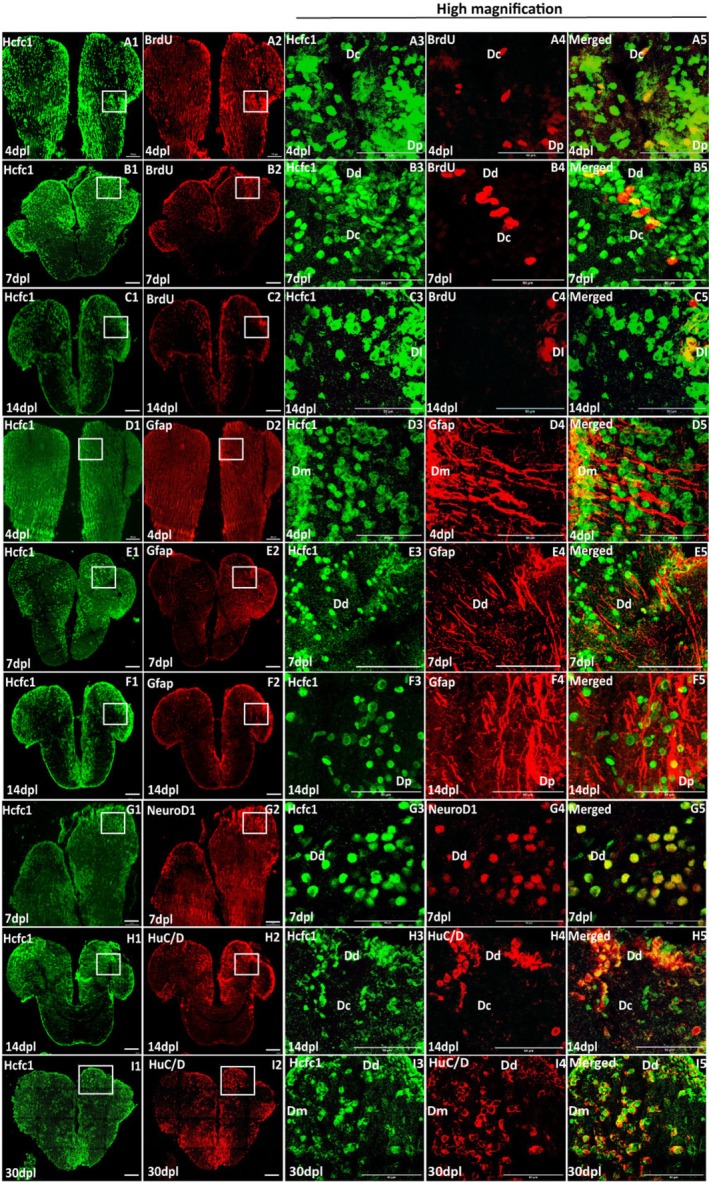
Hcfc1 is upregulated in proliferating cells, glial cells and neuronal cells upon injury‐induced brain regeneration: Panels A, B and C shows immunostaining of regenerating brains (4, 7 and 14 dpl) with anti‐HCFC1 (green) and anti‐BrdU (red). Merged panel shows the co‐localization between the BrdU‐positive cells and cells with increased Hcfc1 expression. Scale bar for panels A1, A2, B1, B2, C1 and C2 is 100 μm and for panels A3, A4, A5, B3, B4, B5, C3, C4 and C5 is 50 μm. Panel D, E and F shows immunostaining of regenerating brains (4, 7 and 14 dpl) with anti‐HCFC1(green), anti‐GFAP (glial cell marker, red). Merged panel shows the co‐localization between the Gfap‐expressing cells (at neurogenic niche and the site of injury) and cells with increased Hcfc1 expression. Scale bar for panels D1, D2, E1, E2, F1 and F2 is 100 μm and for panels D3, D4, D5, E3, E4, E5, F3, F4 and F5 is 50 μm. Panel G shows immunostaining with anti‐HCFC1(green) and anti‐NeuroD1 (neuronal cell marker, red) at 7 dpl. Merged panel shows the co‐localization between the NeuroD1‐expressing cells and cells with increased Hcfc1 expression. Scale bar for panels G1 and G2 is 100 μm and for panels G3, G4 and G5 is 50 μm. Panels H and I show immunostaining with anti‐HCFC1(green) and anti‐HuC/D (neuronal cell marker, red) at 14 and 30 dpl. Merged panel shows the co‐localization between the HuC/D‐expressing cells (at neurogenic niche and the site of injury) and cells with increased Hcfc1 expression. Scale bar for panels H1, H2, I1 and I2 is 100 μm and for panels H3, H4, H5, I3, I4 and I5 is 50 μm. Dd (Dorsal zone of dorsal telencephalic area), Dm (Medial zone of dorsal telencephalic area), Dc (Central zone of dorsal telencephalic area), Dl (later zone of dorsal telencephalic area), Dp (Posterior zone of dorsal telencephalic area).

Further, to explore the role of Hcfc1 in cellular processes during regeneration, we performed immunostaining for proliferation, glial, and neuronal markers. BrdU‐positive cells, during the 4‐h BrdU pulse, were observed at the injury site and in the neurogenic niche, with a marked increase at 4 and 7 dpl, followed by a decrease at 14 dpl. Notably, colocalization of BrdU with Hcfc1‐positive cells suggested that Hcfc1 is expressed in proliferating cells, highlighting its potential role in promoting proliferation during regeneration (Figure 2 panels A1–A5, B1–B5, and C1–C5).

To further investigate the progenitor cell population, we stained brain sections for Gfap, a marker of ependymoglial cells. We observed a marked increase in Gfap expression at 4 dpl in the injured hemisphere in the medial zone of the dorsal telencephalic area (Figure 2 panel D4). This upregulation of Gfap persisted at 7 dpl in both the neurogenic niche and upper parenchyma, reaching its peak at 14 dpl across the parenchyma (Figure 2E,F). As Hcfc1 is expressed in all cells, as seen from its co‐localization with the nuclear stain DAPI at 7 and 14 dpl, all Gfap‐positive cells in these regions are also Hcfc1‐positive (Figure S4A; panels A5 and A10). Co‐staining with another glial progenitor marker, glutamine synthetase, further confirmed Hcfc1 expression in glial progenitors in the injured hemisphere at 7 dpl within the neurogenic niche (Figure S4B). These results suggest that Hcfc1 may play a role in glial progenitor proliferation during the injury response.

To explore the role of Hcfc1 in neuronal regeneration, we examined the expression of NeuroD1, a neuronal cell fate marker, and HuC/D, a pan‐neuronal marker. At 7 dpl, we observed increased expression of NeuroD1 in the pallial region, with co‐localization of Hcfc1 and NeuroD1 at the injury site (Figure 2G). At 14 dpl, HuC/D‐positive neurons were highly upregulated at both the injury site and the neurogenic niche, and these neurons migrated to the parenchyma and periventricular zone at 30 dpl. Co‐localization of HuC/D and Hcfc1 at 14 and 30 dpl further supports the idea that Hcfc1 plays a role in neuronal differentiation and migration during regeneration (Figure 2H,I).

Together, these findings established Hcfc1 and Ogt as key candidates for investigating their role in zebrafish brain regeneration. These results suggest that Hcfc1 appears crucial for glial and neuronal progenitor cell proliferation and differentiation during brain regeneration in zebrafish. The expression levels of HCF‐1 were also explored in a mammalian model (rat) of traumatic brain injury. Increased mRNA levels of *il1b, tnfa*, and *cox2* were observed in the injured brain at 1 and 7 dpi (Figure S5A), signifying an inflammatory response upon injury, which is similar to what is observed in the zebrafish brain upon telencephalic stab‐wound injury [55]. However, upregulation of mRNA levels of *hcfc1* and *ogt* was not observed (Figure S5B). Immunoblotting also showed no significant change in the protein levels of HCF‐1, whereas a slight increase in the proliferation marker PCNA was observed (Figure S5C).

### 2.3 Upregulation of Hcfc1 in Retinal Regeneration

**FIGURE 3:**
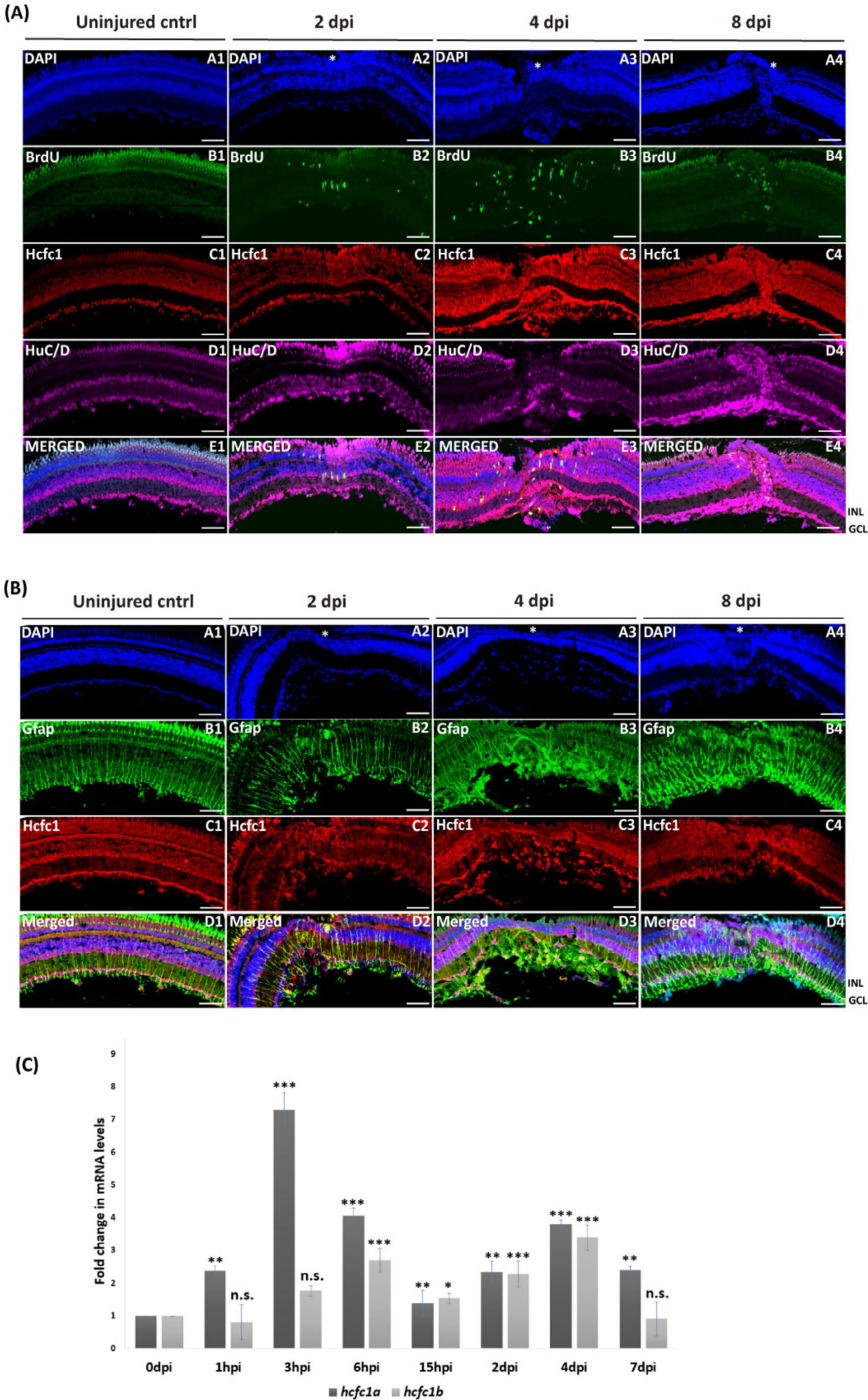
Hcfc1 is upregulated in proliferating cells, glial cells and neuronal cells upon injury‐induced retinal regeneration. (A) Immunostaining with anti‐HCFC1 (red), anti‐BrdU (green), and anti‐HuC/D (magenta) in regenerating retina (2, 4 and 8 dpi) compared to uninjured retina. Counter staining is done with DAPI (blue). Merged panel shows the co‐localization between the BrdU‐positive cells and cells with increased Hcfc1 and HuC/D expression. Scale bar is 10 μm. (B) Immunostaining with anti‐HCFC1(red) and anti‐GFAP (green) in regenerating retina (2, 4 and 8 dpi) compared to uninjured retina. Counter staining is done with DAPI (blue). Merged panel shows the co‐localization between the Gfap‐expressing cells and cells with increased Hcfc1 expression. Scale bar is 10 μm. (C) qRT‐PCR of hcfc1a and hcfc1b in uninjured and injured retina (1 hpi, 3 hpi, 6 hpi, 15 hpi, 2 dpi, 4 dpi and 7 dpi; *n* = 3). Significance is represented as * for *p*‐value < 0.05, ** for *p*‐value < 0.01 and *** for *p*‐value < 0.001 for all the quantifications. INL, inner nuclear layer; GCL, ganglion cell layer; white asterisk represents the site of injury.

To further explore the role of Hcfc1 and Ogt in regeneration, we extended our investigations to the zebrafish retina, an additional model of tissue regeneration. Similar to the brain injury model, we examined the expression dynamics of Hcfc1 during retinal regeneration. Immunostaining for the proliferation marker BrdU revealed a significant increase in BrdU‐positive cells at the injury site in 2 days post‐injury (2 dpi) and 4 dpi retina, which declined by 8 dpi (Figure 3A,B). Notably, these proliferating cells co‐localised with cells expressing elevated levels of Hcfc1, suggesting a link between Hcfc1 expression and the proliferative response (Figure 3A panel B4, C4 and E4).

Additionally, we assessed the expression of neuronal (HuC/D) and glial (Gfap) markers and observed similar co‐localization of these cells with Hcfc1 expression as was seen in brain regeneration (Figure 3A panel C, D, E, and Figure 3B). This indicates that Hcfc1 may be crucial for brain and retinal regeneration, further implicating its broader role in regeneration across zebrafish tissues. This prompted us to look into the interacting partners of Hcfc1 during retinal regeneration. In this regard, a co‐immunoprecipitation assay was performed in control retinae and regenerating retinae at 2 dpi. Our mass spectrometry analysis revealed distinct Hcfc1 interacting partners during regeneration as compared to the control retina. The Hcfc1 interacting proteins in the regenerating retina were identified to be involved in processes such as metabolic and mitochondrial support, neurogenesis and proliferation, inflammation, vascular remodelling, and cell cycle and DNA replication (Figure S6A and Tables S2 and S3). Further, gene ontology enrichment analysis of Hcfc1 interacting proteins in control retina revealed enriched terms such as Peptidyl lysine methylation, Mesoderm development, Regulation of chromosome organisation, etc., and terms like Apoptotic mitochondrial change, Regulation of vasculature development, Protein‐containing complex assembly, etc., were enriched upon gene ontology enrichment analysis of regenerating retina (Figure S6B,C).

Further, we conducted qPCR analysis to assess the temporal expression of both *hcfc1a* and *hcfc1b* in the regenerating retina. The results showed an initial increase in the expression of both paralogs at 3 h post‐injury (hpi) and 6 hpi, followed by a dip at 15 hpi. Interestingly, expression levels surged again at 2 and 4 dpi, consistent with the proliferation peaks observed during these time points (Figure 3C). These results suggest that the upregulation of Hcfc1 during retinal regeneration mirrors its role in brain regeneration, reinforcing the idea that Hcfc1 is a key player in driving the regenerative response in zebrafish tissues.

### 2.4 Investigating the Role of Hcfc1 in Regeneration Through Loss‐Of‐Function Approaches

We employed targeted loss‐of‐function strategies to better understand the functional significance of Hcfc1 in zebrafish regeneration. Given the established importance of Hcfc1 in development [50, 51] and its potential role in regeneration, we explored the consequences of its depletion in both embryonic and regenerative contexts. HCF‐1 is known to be crucial for embryonic development, with its knockout being lethal in murine embryos [56, 57]. To assess its role in zebrafish development, we performed morpholino (MO)‐mediated knockdown of both *hcfc1a* and *hcfc1b* paralogs in zebrafish embryos. MO‐injected embryos exhibited a range of developmental defects, including bent body axis, curved tail, CNS deformities, and cardiac edema [51] (data not shown).

**FIGURE 4:**
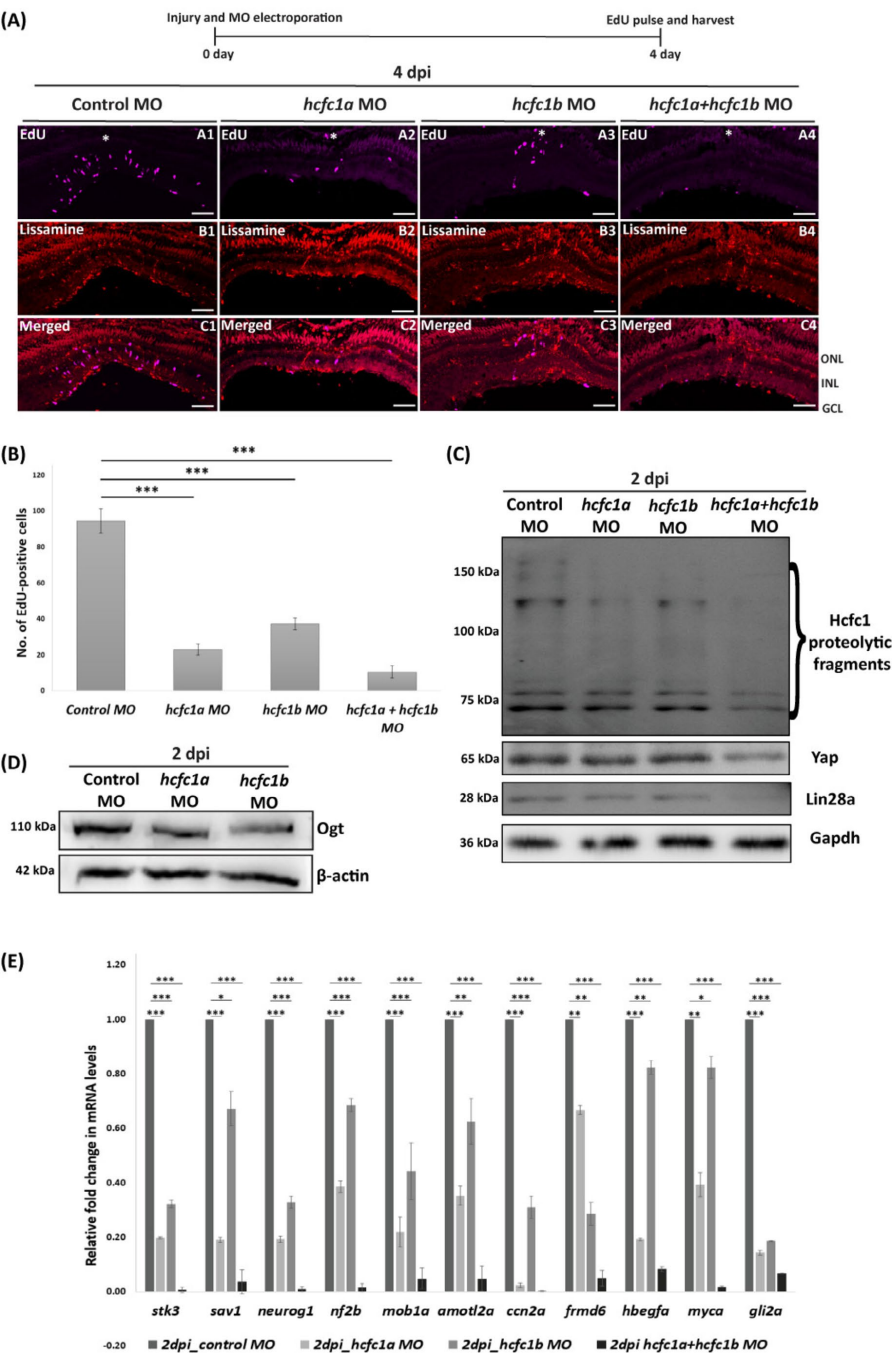
Mo‐induced Hcfc1a and Hcfc1b knockdown affects proliferation and Hippo/Yap signalling pathway during retinal regeneration. (A) Immunostaining with anti‐EdU (magenta) in 4 dpi regenerating retina injected with control morpholino and morpholino against hcfc1a (0.5 mM), hcfc1b (0.5 mM) and both combined (0.5 mM). Panel B shows the distribution of morpholinos which are lissamine tagged (red). Scale bar is 10 μm. INL, inner nuclear layer; GCL, ganglion cell layer; ONL, outer nuclear layer; white asterisk represents the site of injury. (B) Quantification of EdU‐positive cells at 4 dpi regenerating retina injected with control morpholino and morpholino against hcfc1a, hcfc1b and both combined (*n* = 11). (C) Immunoblotting with ant‐HCFC1, anti‐YAP and anti‐Lin28a in 2 dpi regenerating retina injected with control morpholino and morpholino against hcfc1a (0.5 mM), hcfc1b (0.5 mM) and both combined (0.5 mM). Anti‐Gapdh was used as loading control. (D) Immunoblotting with anti‐OGT in 2 dpi regenerating retina injected with control morpholino and morpholino against hcfc1a (0.5 mM) and hcfc1b (0.5 mM). Anti‐β‐Actin was used as loading control. (E) qRT‐PCR of Hippo/Yap pathway proteins and its downstream targets in 2 dpi regenerating retina injected with control morpholino and morpholino against hcfc1a (0.5 mM), hcfc1b (0.5 mM) and both combined (0.5 mM) (*n* = 3). Significance is represented as * for *p*‐value < 0.05, ** for *p*‐value < 0.01 and *** for *p*‐value < 0.001.

Due to technical constraints posed by cerebroventricular MO microinjections, we focused on retinal regeneration, where MOs targeting *hcfc1a* and *hcfc1b* were injected into injured zebrafish retina. Functional analysis revealed a marked reduction in EdU‐positive proliferating cells at 4 dpi in *hcfc1a‐, hcfc1b‐*, and both *hcfc1a‐* and *b*‐depleted retina. The reduction was more pronounced upon *hcfc1a* knockdown than *hcfc1b*, with a drastic decline observed upon combined loss of both paralogs, indicating their collective necessity for efficient retinal regeneration (Figure 4A,B). Their knockdown efficiency was validated via immunoblotting, which showed a significant reduction in Hcfc1 levels at 2 dpi (Figure 4C). Notably, combined knockdown of both paralogs resulted in a more pronounced downregulation compared to individual knockdowns (Figure 4C). Furthermore, knockdown of *hcfc1a* and *hcfc1b* led to a reduction in Ogt levels during retinal regeneration at 2 dpi, suggesting that Ogt levels are probably regulated by Hcfc1 (Figure 4D).

### 2.5 Pharmacological Inhibition of the Hcfc1‐Ogt Axis During Brain and Retinal Regeneration

**FIGURE 5:**
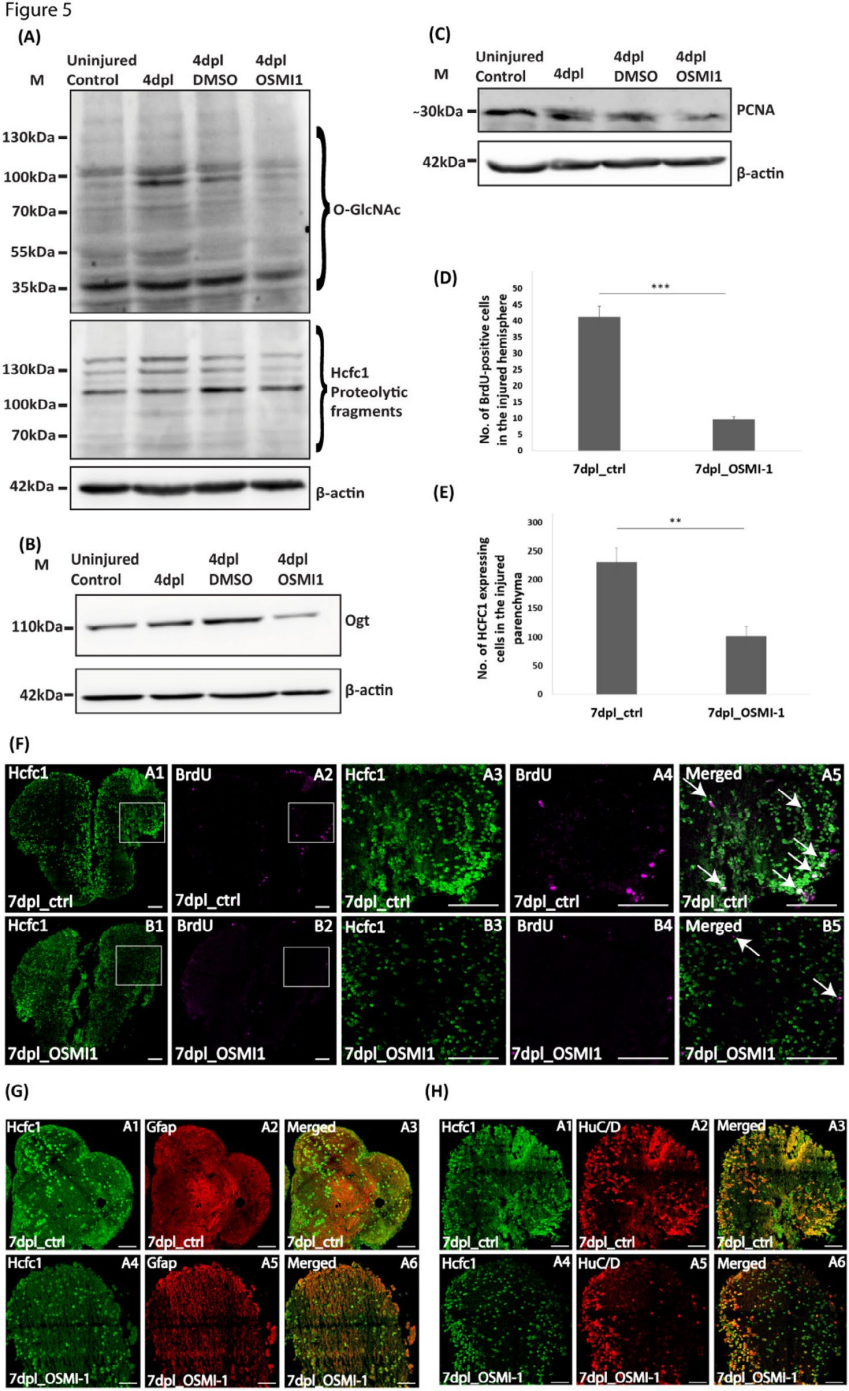
Effect of Ogt activity inhibition on Hcfc1 expression and regeneration in the brain. (A) Immunoblot of regenerating brain (DMSO treated) and OSMI‐1 (50 μM) treated brain at 4 dpl with anti‐*O*‐GlcNac (RL2) and anti‐HCFC1, (B) Immunoblot of regenerating brain (DMSO treated) and OSMI‐1 (50 μM) treated brain at 4 dpl with anti‐OGT (proliferation marker), (C) Immunoblot of regenerating brain (DMSO treated) and OSMI‐1 (50 μM) treated brain at 4 dpl with anti‐PCNA. Anti‐β‐Actin was used as a loading control for the three immunoblots. (D) Quantification of BrdU‐positive cells in the injured hemisphere of regenerating control (DMSO treated) and OSMI‐1 (50 μM) treated brains at 7 dpl (*n* = 3). (E) Quantification of Hcfc1‐epressing cells in the parenchyma of the injured hemisphere of regenerating control (DMSO treated) and OSMI‐1 (50 μM) treated brains at 7 dpl (*n* = 3). Significance is represented as ** for *p*‐value < 0.01 and *** for *p*‐value < 0.001. (F) Immunostaining of regenerating control brain (DMSO treated) and OSMI‐1 (50 μM) treated brains with anti‐HCFC1(green) and anti‐BrdU (proliferation marker, magenta) at 7 dpl. Merged panel shows co‐localization with BrdU‐positive cells and Hcfc1 expressing cells. Scale bar for panels A1, A2, B1 and B2 is 100 μm and for panels A3–A5 and B3–B5 is 50 μm. (G) Immunostaining of regenerating control brain (DMSO treated) and OSMI‐1 (50 μM) treated brains at 7 dpl with anti‐HCFC1(green) anti‐GFAP (red). Merged panels A3 and A6 show co‐localization with Gfap expressing cells and Hcfc1 expressing cells in the injured hemisphere of the regenerating brain. Scale bar is 50 μm. (H) Immunostaining of regenerating control brain (DMSO treated) and OSMI‐1 (50 μM) treated brains at 7 dpl with anti‐HCFC1(green) anti‐HuC/D (red). Merged panels A3 and A6 show co‐localization with HuC/D expressing and Hcfc1 expressing cells in the injured hemisphere of the regenerating brain. Scale bar is 50 μm.

To further explore the Hcfc1‐Ogt axis, we employed OSMI‐1 [58, 59], a pharmacological inhibitor of *O*‐GlcNAc transferase (Ogt), which regulates Hcfc1 activity through glycosylation. The efficacy of OSMI‐1 treatment was confirmed by immunoblot analysis of RL2 (a marker for global *O*‐GlcNAcylation), which showed decreased *O*‐GlcNAcylation levels post‐treatment (Figure 5A and Figure S7A). Notably, a corresponding reduction in Hcfc1 expression was also observed, confirming the interdependence of Ogt activity and Hcfc1 stability (Figure 5A). Ogt expression levels were also reduced following OSMI‐1 treatment (Figure 5B), contrary to previous reports with tissue culture cells [58, 60, 61]. Inhibition of Ogt activity resulted in a marked reduction in cellular proliferation, as evidenced by decreased PCNA expression in immunoblots (Figure 5C). Immunostaining analysis further revealed fewer BrdU‐positive cells in the injured parenchyma (Figure 5D,F; compare panels A1‐A5 to B1‐B5) and a significant reduction in Hcfc1‐expressing cells at the injury site in OSMI‐1 treated fish (Figure 5E,F, compare panels A3 and B3). Additionally, it was revealed that in parallel to Hcfc1 reduction, fewer Gfap‐positive glial progenitors were found in the injured hemisphere at 7 dpl in OSMI‐1 treated fish compared to controls (Figure 5G, compare A2 and A5). Similarly, HuC/D‐positive neuronal cells were also reduced, highlighting impaired neurogenesis and a failure to repopulate the injured brain region (Figure 5H, compare A2 and A5). The observed reduced cellular accumulation at the injury site suggested impaired regenerative capacity. Importantly, TUNEL assays did not show significant differences in apoptosis levels between the injured hemispheres of regenerating control and OSMI‐1 treated brains, indicating that the observed reduction in cell number was primarily due to decreased proliferation rather than increased cell death (Figure S7B,C).

**FIGURE 6:**
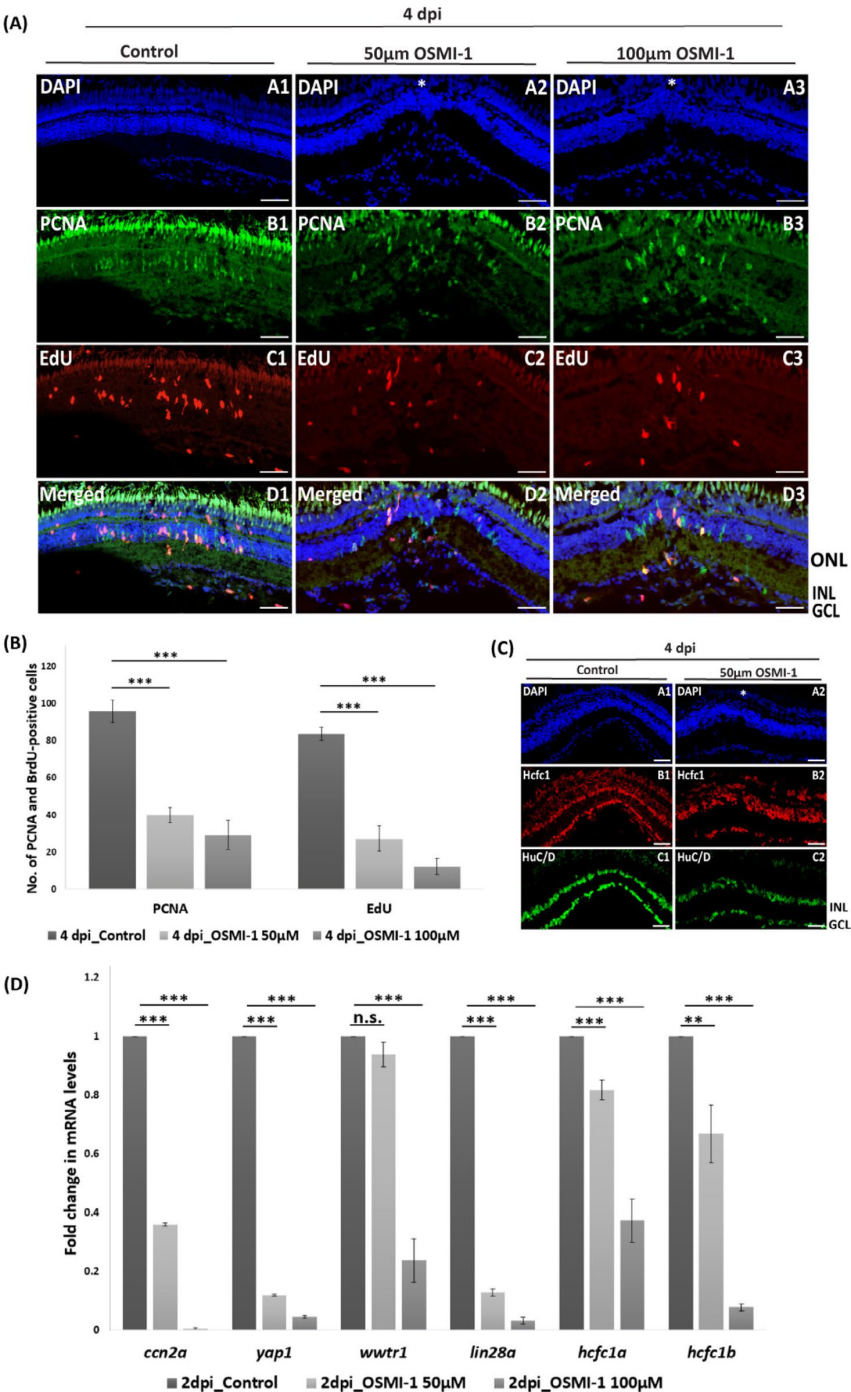
Effect of Ogt activity inhibition on Hcfc1 expression and regeneration in the retina. (A) Immunostaining of regenerating control retina (DMSO treated) and OSMI‐1 (50 μM and 100 μM) treated retina at 4 dpi with anti‐EdU (red) and anti‐PCNA (green). Counterstaining is done with DAPI (blue). Scale bar is 10 μm. (B) Quantification of PCNA‐positive and EdU‐positive cells in regenerating control retina (DMSO treated) and OSMI‐1 (50 and 100 μM) treated retina at 4 dpi (*n* = 5). (C) Immunostaining of regenerating control retina (DMSO treated) and OSMI‐1 (50 μM) treated retina at 4 dpi with anti‐HCFC1(red) and anti‐HuC/D (green). Counterstaining is done with DAPI (blue). Scale bar is 10 μm. (D) qRT‐PCR of *hcfc1a, hcfc1b, yap1, wwtr1* and downstream targets of Yap in regenerating control retina (DMSO treated) and OSMI‐1 (50 and 100 μM) treated retina at 2 dpi (*n* = 3). Significance is represented as ** for *p*‐value < 0.01, *** for *p*‐value < 0.001 and n.s. for *p*‐value > 0.05. INL, inner nuclear layer; GCL, ganglion cell layer; ONL, outer nuclear layer; white asterisk represents the site of injury.

Analogous experiments in the regenerating zebrafish retina showed a similar trend upon OSMI‐1 treatment. Immunostaining for proliferation markers EdU and PCNA at 4 dpi revealed a significant reduction in proliferating cells and Hcfc1 expression compared to untreated controls (Figure 6A,B). Additionally, the expression of HuC/D, a marker for differentiated neurons, was reduced in OSMI‐1‐treated retinae, indicating impaired neuronal regeneration (Figure 6C). The qPCR analysis further confirmed the downregulation of *hcfc1a* and *hcfc1b* transcript levels in the injured retina (Figure 6D). These results collectively suggest that the Hcfc1‐Ogt axis plays a critical role in both brain and retinal regeneration in zebrafish.

### 2.6 Investigating the Impact of Hcfc1 and Ogt on Hippo/Yap signalling

We hypothesized that since Ogt catalyzes *O*‐GlcNAcylation of a vast array of proteins, and Hcfc1 is known to bind to the promoter regions of approximately 5000 genes [46], any dysregulation in their functions could significantly impact multiple signalling pathways crucial for regeneration. To explore this, we assessed the expression of key genes from essential regenerative signalling pathways using qPCR in regenerating DMSO control and OSMI‐1‐treated regenerating retina at 2 dpi. Interestingly, we observed a downregulation in the transcript levels of key Hippo/Yap pathway components, namely *yap1* and *wwtr1*, along with their downstream targets *ccn2a* and *lin28a* (Figure 6D). Our results in brain regeneration also showed that upon OSMI‐1‐mediated Ogt activity inhibition, levels of phosphorylated (inactive) Yap increase at 7 dpl compared to the regenerating control (Figure S7D,E).

To explore this connection in zebrafish regeneration, we analysed Hcfc1 knockdown (MO‐injected) retinal samples via immunoblotting for Yap and Lin28a, both of which showed reduced expression compared to controls (Figure 4C). These findings were further validated through qPCR, confirming the downregulation of downstream Yap targets. Interestingly, we also observed a significant reduction in the expression of upstream Hippo/Yap pathway regulators, including *stk3, sav1, nf2b, mob1a, amot1a, and frmd6* (Figure 4E). This unexpected finding suggests that, despite the downregulation of *yap1*, the upstream regulatory components of the Hippo/Yap pathway are also affected. These results hint at the possibility that, during regeneration, Yap levels may be regulated via Hippo‐independent mechanisms, potentially involving Hcfc1‐mediated epigenetic regulation, as previously suggested in Drosophila [62].

### 2.7 Yap Plays an Important Role During Brain and Retinal Regeneration

Given that *yap1* mRNA levels were altered during retinal regeneration upon both OSMI‐1 treatment and Hcfc1 knockdown, we aimed to investigate the role of Yap during brain regeneration. Yap has been previously shown to play an essential role in zebrafish development and regeneration [63, 64, 65]. Considering its established roles in development and retinal regeneration, we examined Yap expression in zebrafish brain regeneration.

**FIGURE 7:**
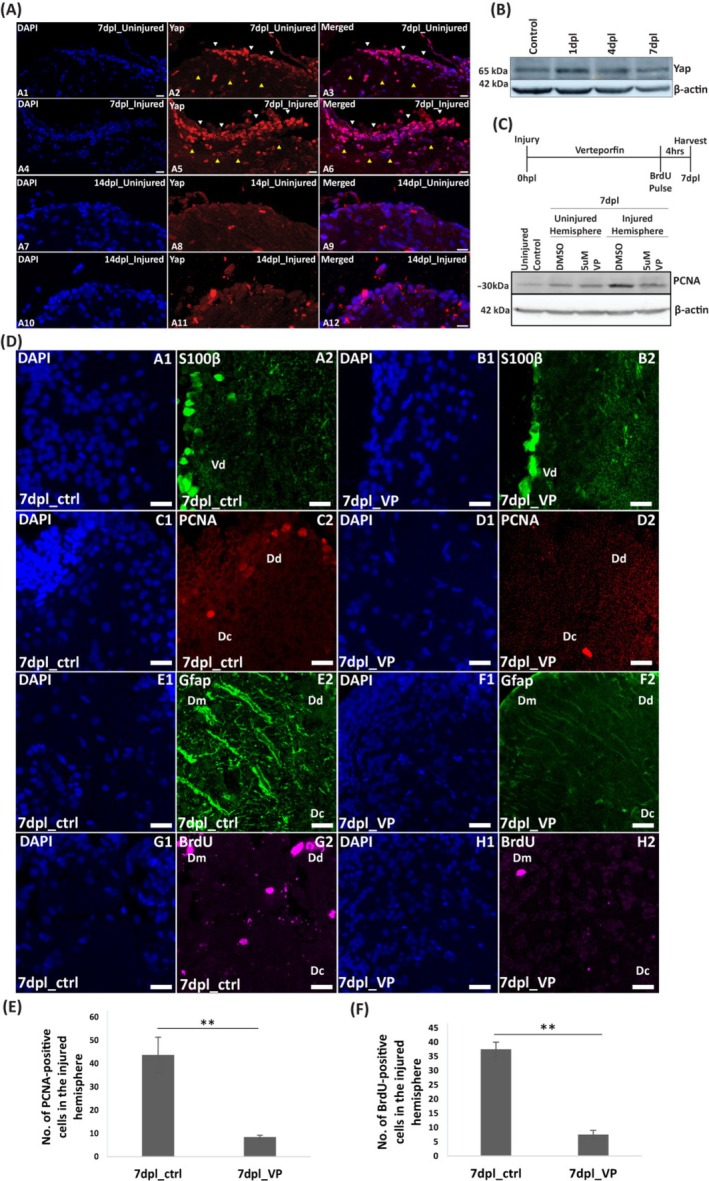
Yap is upregulated during brain regeneration in zebrafish and its inhibition affects brain regeneration. (A) Immunostaining of regenerating brains at 7 and 14 dpl with anti‐YAP (red) and counterstained with DAPI (blue). Scale bar is 50 μm. (B) Immunoblot of regenerating brains (uninjured control, 1, 4 and 7 dpl) with anti‐YAP. Anti‐β‐Actin was used as a loading control. (C) Immunoblot of regenerating brain and Verteporfin (5 μM) treated brain at 7 dpl with anti‐PCNA and anti‐β‐Actin was used as a loading control. (D) Immunostaining of regenerating control brain (DMSO treated) and Verteporfin (5 μM) treated brains at 7 dpl with anti‐s100β (green, panel A and B), anti‐PCNA (red, panel C and D), anti‐GFAP (green, panel E and F) and anti‐BrdU (magenta, panel G and H). Scale bar is 50 μm. (E) Quantification of no. of PCNA‐positive cells in the injured telencephalic hemisphere of DMSO control and Verteporfin treated 7 dpl brains (*n* = 3). (F) Quantification of no. of BrdU‐positive cells in the injured telencephalic hemisphere of DMSO control and Verteporfin treated 7 dpl brains (*n* = 3). Significance is represented as ** for *p*‐value < 0.01. Vd (Dorsal nucleus of ventral telencephalic area), Dd (Dorsal zone of dorsal telencephalic area), Dm (Medial zone of dorsal telencephalic area), Dc (Central zone of dorsal telencephalic area), Dl (later zone of dorsal telencephalic area).

Immunostaining showed increased Yap expression predominantly in neurogenic niches such as the laterodorsal and posterior zones of the dorsal telencephalon (Dl and Dp) and the periventricular region of the dorsal telencephalon at 7 dpl in the injured hemisphere compared to the uninjured side (Figure 7A, compare A2 and A5). Nuclear localization of Yap was predominant in neurogenic niches (Figure 7A, A6, white arrowheads), while a subset of cells exhibited cytoplasmic localization (Figure 7A, A6, yellow arrowheads). By 14 dpl, Yap expression remained elevated, but the majority of cells displayed cytoplasmic localization, suggesting inactivation of Yap signalling (Figure 7A, A10–A12). Immunoblotting also revealed that total Yap levels increased during the early regenerative response, with peak expression observed at 1 day post‐lesion (1 dpl) and sustained upregulation up to 7 dpl (Figure 7B). Brain sections were co‐immunostained with progenitor glial marker glutamine synthetase and S100β, neuronal marker HuC/D and BrdU to understand the cellular context of Yap expression. We observed co‐localization of nuclear Yap with glutamine synthetase and S100β‐positive progenitor cells at 4 and 7 dpl respectively, suggesting that Yap is active and upregulated in glial progenitors (Figure S8; panel A1–A5 & C1–C4). Further, co‐localization of Yap was also observed with HuC/D and BrdU‐positive proliferating cells (Figure S8 B1–B5 & D1–D4) at 4 dpl in the injured hemisphere.

Further, to assess the impact of Yap inhibition on brain regeneration, we utilised the pharmacological inhibitor Verteporfin. Verteporfin suppresses Yap activity by promoting its cytoplasmic sequestration through the upregulation of 14–3‐3 proteins, which prevents Yap nuclear translocation and subsequent transcriptional regulation [66]. Immunoblotting of the proliferation marker PCNA showed a significant reduction in expression in the Verteporfin‐treated injured telencephalic hemisphere compared to the untreated injured control and uninjured hemisphere controls (Figure 7C). Further, immunostaining with proliferation markers PCNA and BrdU in Verteporfin‐treated samples revealed a decrease in the number of PCNA‐positive (Figure 7D, compare C2 and D2 & 7 E) and BrdU‐positive cells (Figure 7D, compare G2 and H2 & 7 F) in the injured hemisphere compared to the untreated regenerating control. In addition, immunostaining with glial markers S100β and Gfap demonstrated reduced expression levels in the Verteporfin‐treated injured hemisphere compared to the untreated control (Figure 7D, compare A2 and B2 for S100β; E2 and F2 for Gfap).

These results suggest that Yap is crucial during the early phase of injury‐induced regenerative response and is vital in regulating the proliferation of Gfap and S100β‐positive progenitor glial cells. Loss of Yap function leads to impaired proliferation and accumulation of regenerative cells, highlighting its essential role in regeneration.

### 2.8 *yap5SA* mRNA Overexpression Rescues the Effects of Hcfc1 Loss During Regeneration

Having established the critical role of Yap in regeneration and its downregulation upon Ogt inhibition and Hcfc1 loss, we hypothesized a potential Hcfc1‐Ogt‐Yap axis driving the regenerative process. To explore this, we sought to determine whether overexpression of Yap could counteract the effects of Hcfc1 loss and Ogt inhibition.

For this purpose, we utilised a constitutively active Yap mutant, *yap5SA*, in which five serine residues were substituted with alanine to prevent phosphorylation‐induced degradation, leading to persistent YAP activity [67]. *yap5SA* mRNA was injected into the regenerating retina, alongside treatments that included OSMI‐1 (an OGT inhibitor) and morpholino (MO) injections targeting *hcfc1a*, *hcfc1b*, or both *hcfc1a* and *hcfc1b* combined with OSMI‐1 prior to injury.

**FIGURE 8:**
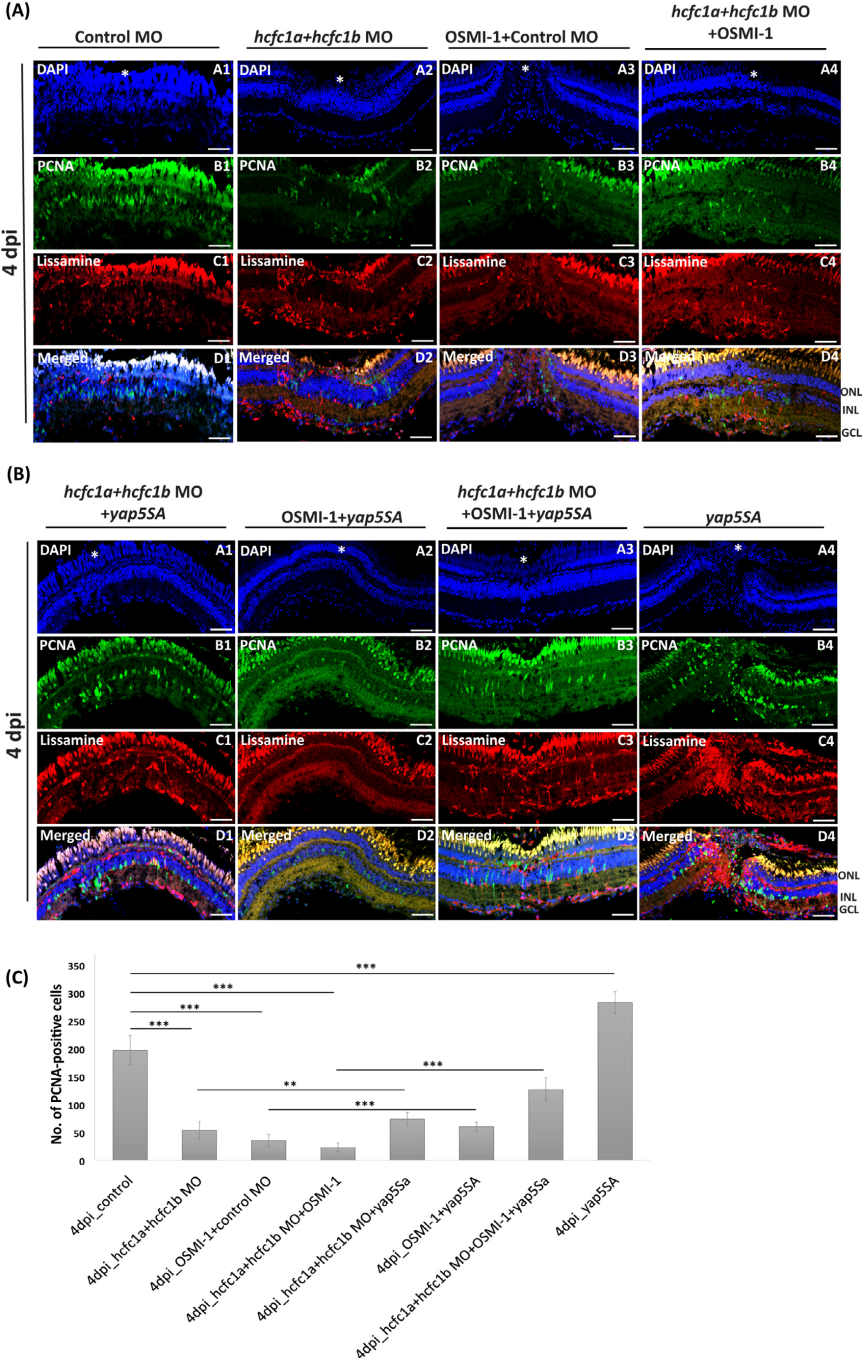
Yap overexpression rescues the proliferation effects of Hcfc1 knockdown and Ogt activity inhibition during retinal regeneration. (A) Immunostaining of regenerating retina (control, Hcfc1 knockdown (0.5 mM each MO) and OSMI‐1 (50 μM) treated) at 4 dpi with anti‐PCNA (green). Counterstaining is done with DAPI (blue). Scale bar is 10 μM. Panel C shows the distribution of morpholinos which are lissamine tagged (red). (B) Immunostaining of regenerating retina with *yap5SA* mRNA injection (control, Hcfc1 knockdown (0.5 mM each MO) and OSMI‐1 (50 μM) treated) at 4 dpi with anti‐PCNA. Counterstaining is done with DAPI. Scale bar is 10 μM. Panel C shows the distribution of morpholinos which are lissamine tagged (red). (C) Quantification of PCNA‐positive cells in regenerating retina with Hcfc1 knockdown, OSMI‐1 treatment with and without *yap5SA* mRNA injection (*n* = 9). Significance is represented as ** for *p*‐value < 0.01 and *** for *p*‐value < 0.001. INL, inner nuclear layer; GCL, ganglion cell layer; ONL, outer nuclear layer; white asterisk represents the site of injury.

One of the common effects of both OSMI‐1 treatment and morpholino‐mediated loss of *hcfc1* was decreased proliferation; therefore, we analysed if Yap overexpression would rescue the defects in proliferation. At 4 dpi, we observed that Yap overexpression could indeed rescue the defects in proliferation. Upon Yap overexpression, the number of PCNA‐positive cells increased in both OSMI‐1 treated and *hcfc1a* and *hcfc1b* MO injected regenerating retinal samples (Figure 8A,B). The increase in the number of PCNA‐positive cells was greater in yap5SA overexpression combined with *hcfc1a* and *hcfc1b* loss compared to yap5SA overexpression combined with OSMI‐1 treated regenerating samples (Figure 8B compare panel B1 and B2). The highest increase was observed in *yap5SA* overexpression combined with *hcfc1 (hcfc1a and hcfc1b)* loss with OSMI‐1 treatment (Figure 8C), suggesting that the Yap rescue effect is more prominent when there is an increased loss of *hcfc1* function, as OSMI‐1 treatment also reduces Hcfc1 levels during regeneration.

These results highlight the role of a novel Hcfc1‐Ogt‐Yap axis in regulating regeneration. This further prompted us to investigate if Yap also controls the levels of Hcfc1. Interestingly, upon morpholino‐mediated *yap1* knockdown in the regenerating retina at 2 dpi, decreased mRNA levels of both *hcfc1a* and *hcfc1b* were observed along with the expected decreased mRNA levels of *wwtr1, yap1*, and its downstream targets *ccn2a* and *lin28a* (Figure S9A). This was also observed in vitro through cell culture studies using a mammalian neuroblastoma cell line, SH‐SY5Y, wherein, upon verteporfin‐mediated pharmacological inhibition of Yap, there was a significant reduction in Hcfc1 levels (Figure S9B). These results hint towards a feedback loop suggesting that Yap expression might also regulate Hcfc1.

## 3 Discussion and Conclusion

Tissue regeneration in the CNS represents a biologically complex yet clinically critical process. In this study, we have identified Host Cell Factor‐1 (Hcfc1) and *O*‐GlcNAc transferase (Ogt) as important regulators of neural regeneration, mediated partly by their influence on the Hippo/Yap signalling pathway. Our findings build upon prior research and offer novel insights into the molecular landscape of CNS regeneration.

The functional importance of HCFC1 as a transcriptional co‐regulator and its interaction with OGT has been well documented in developmental and metabolic contexts [68, 69, 70]. Our findings confirm its crucial role in CNS regeneration and reveal that its knockdown and pharmacological inhibition of Ogt impair regenerative processes in the zebrafish brain and retina. Additionally, our results highlight the disruption of Hippo/Yap signalling components, particularly Yap expression, followed by the loss of Hcfc1 and Ogt activity. It has been shown that mutations in zebrafish ortholog *hcfc1a* disrupt the levels of 14–3‐3β/α, suggesting a possible regulation of Yap levels by modulating 14–3‐3β/α levels due to Hcfc1 loss during retinal regeneration [54]. Our co‐immunoprecipitation assay showed distinct interacting partners of Hcfc1 during retinal regeneration at 2 dpi. One of the interacting partners revealed was β‐catenin. Interestingly, it has been previously known that in murine epiblast‐specific knockout of *hcfc1*, levels of nuclear β‐catenin were reduced [57]. Therefore, the interaction of β‐catenin with Hcfc1 during retinal regeneration highlights an interesting avenue in the field.

A critical aspect of regenerative processes that has caught attention is the role of glycosylation as a downstream consequence of metabolic reprogramming. During tissue regeneration, cells often exhibit a metabolic shift from oxidative phosphorylation to aerobic glycolysis. This metabolic reprogramming facilitates the production of metabolites essential for glycosylation, which, in turn, governs various cellular processes, which has been shown in the case of zebrafish heart and tail regeneration [71, 72, 73]. Our findings corroborate these observations, as we demonstrate that Hcfc1 and Ogt, critical regulators of *O*‐GlcNAcylation, are indispensable for neural regeneration in the zebrafish retina and brain. Loss of Hcfc1 and Ogt activity disrupts the Hippo/Yap pathway and likely interferes with glycosylation‐dependent signalling mechanisms. Together, these insights highlight a conserved mechanism wherein glycosylation serves as a crucial modulator of regeneration. Understanding how glycosylation integrates with transcriptional co‐regulators such as Hcfc1 can provide additional cues to understand this process.

The Hippo pathway, a conserved regulator of tissue homeostasis and organ size, is recognised for its role in regeneration [74, 75, 76]. Previous studies have shown that Yap/Taz activity facilitates regenerative responses in zebrafish across multiple tissues [63]. Our data confirm that Yap activation is necessary for neural regeneration, as pharmacological inhibition of Yap using verteporfin significantly impaired regeneration. Furthermore, overexpression of a constitutively active Yap5SA construct rescued proliferation defects induced by reduced Hcfc1 and Ogt. Interestingly, *yap1* knockdown during retinal regeneration led to reduced transcript levels of *hcfc1a* and *hcfc1b*, suggesting a possible feedback system and establishing a mechanistic link between these factors and Hippo/Yap pathway activity.

The role of OGT in linking metabolic signals to cellular processes via *O*‐GlcNAcylation has been previously demonstrated in the context of the Hippo pathway. Studies have shown that YAP is directly *O*‐GlcNAcylated by OGT, disrupting its interaction with upstream kinases and enhancing its transcriptional activity [77]. Our findings confirm the relevance of this mechanism in CNS regeneration, as Ogt inhibition disrupted Yap levels and regenerative capacity. Both brain and retina exhibit a dependence on Yap activity to orchestrate regenerative responses post‐injury, as well as metabolic shifts [63, 73, 78].

Finally, Host Cell Factor‐1 (HCF‐1) emerges as a critical regulator in various cellular contexts, with roles that are context‐dependent and diverse across tissues and species. In the murine brain, conditional knockout using Nkx2.1‐Cre revealed that HCF‐1 is essential for the survival of GABAergic interneurons and glia, and their numbers decrease upon loss of HCF‐1 due to increased cell death [49]. Intriguingly, our data suggest that loss of Hcfc1 expressing cells via OSMI‐1 mediated Ogt activity inhibition causes increased cell death only in the uninjured telencephalic hemisphere of the zebrafish brain. In contrast, we do not observe any significant increase in cell death in the regenerating telencephalic hemisphere, further emphasizing that Hcfc1 activity depends on the cellular microenvironment. Further, upon traumatic brain injury in rats, inflammation markers are upregulated [55] as also shown in our TBI experiments, which is similar to what is observed in the zebrafish brain and retina upon injury‐induced regeneration. Interestingly, our results show no upregulation of *hcfc1* and *ogt*, along with no significant difference in HCF‐1 protein levels at 7 dpi upon TBI, probably owing to a non‐permissive environment. These results further underline that Hcfc1 exerts its regeneration‐specific role in the zebrafish brain and retina due to a specific permissive cellular microenvironment.

Furthermore, in zebrafish, Hcfc1a and Hcfc1b have distinct but overlapping roles, with Hcfc1b being indispensable for craniofacial development [50, 51, 79]. Our findings connect these developmental roles to regeneration, where many processes initially characterised during development are repurposed. The parallels between its developmental and regenerative functions underscore its fundamental role in cellular plasticity and its capacity to regulate transcriptional programs essential for injury repair. Additionally, previous reports in Drosophila have linked HCF‐1 to Hippo signalling, aligning with its observed influence on Yap stability in our current work [62]. These data suggest that HCF‐1 operates as a central integrator of metabolic and signalling pathways, making it indispensable for tissue development and regeneration.

Further, loss of Hcfc1 reduces Ogt levels, consistent with prior reports demonstrating that HCF‐1 is essential for maintaining OGT stability [44, 80]. Reduced Ogt may, in turn, decrease Yap *O*‐GlcNAcylation, which has been shown to promote nuclear translocation and activation of Yap, thus making Yap more susceptible to phosphorylation. Phosphorylated Yap remains inactive in the cytoplasm, thereby limiting its transcriptional activity [77].

Therefore, we propose that during regeneration, Hcfc1 and Ogt are mutually important for maintaining each other's stability and function, with Ogt playing a critical role in regulating Yap activity through *O*‐GlcNAcylation. This model provides a framework that links Hcfc1 loss to impaired Yap activation via modulation of Ogt levels and highlights a regulatory axis potentially critical for regenerative processes in zebrafish CNS. Future work will be necessary to experimentally validate this mechanism and delineate the precise molecular interactions involved.

In conclusion, our study reveals Hcfc1 and Ogt as essential regulators of neural regeneration in the zebrafish brain and retina. We provide mechanistic insights into their roles in regenerative biology by linking these factors to the Hippo/Yap pathway. These findings contribute to the broader understanding of CNS regeneration and highlight potential avenues for therapeutic modulation of regenerative processes in vertebrates. Future studies should focus on delineating the cell type‐specific roles of Hcfc1 and Ogt and exploring their therapeutic potential in mammalian models.

## 4 Methods and Materials

### 4.1 Ethics Statement

The research adhered to rigorous ethical standards and principles of inclusion to ensure both the animals' welfare and the study's scientific integrity. Experimental protocols were reviewed and approved by the Institute Animal Ethical Committee of IISER Mohali (IISERM/SAFE/PRT/2024/031). Every effort was made to minimise the number of zebrafish used, ensuring that only the necessary amount was employed to yield reliable data for statistical analysis. Additionally, zebrafish of similar age and developmental stage were selected to maintain consistency and accuracy in the experimental outcomes.

Male Wistar rats of weights ranging from 230 to 250 g were acquired from the Central Animal House Facility (CAHF) after approval of experimental procedures for the animal protocol number 1745 by the Institutional Animal Ethics Committee (CPCSEA, Registration number: 173/GO/ReBi/2000/CPCSEA) of Jamia Hamdard University, New Delhi, India. Upon acquisition, 2 animals were housed per cage at standard conditions in an air‐filtrated unit, as reported previously [81]. The animals were provided with the standard diet certified and approved by the CAHF for all rodents and purified water *ad libitum* throughout the course of the study.

### 4.2 Zebrafish Maintenance and Induction of Stab Wound Injury

Zebrafish were housed in the fish facility at a 25°C–28°C temperature range, with a 14‐h light/10‐h dark cycle. The fish used in these studies were between 5 and 8 months old. To induce a telencephalic stab wound injury for brain regeneration studies [53], zebrafish were briefly anaesthetised using MS222 (Ethyl 3‐aminobenzoate methanesulfonic acid; Catalogue no. 20660, Cayman). A 30G needle was then introduced perpendicularly to the skull to induce a stab injury on the right hemisphere of the telencephalon. The contralateral left hemisphere served as the control for assessing regenerative activity. The retinal injury was performed following the method described previously [23]. After the injury, the fish were returned to the system water and sacrificed at different time points. The brain and retina were subsequently isolated and processed for downstream analysis.

### 4.3 Traumatic Brain Injury Surgery in Rats

TBI was induced by the controlled cortical impact injury method using standardised equipment as described previously with minor modifications as necessitated by laboratory conditions [82]. Briefly, the experimental animals were anaesthetised by intraperitoneal (i.p.) administration of Xylazine (10 mg/kg b. wt.) and Ketamine (75 mg/kg b. wt.) prior to surgery. After that, the animal was put on a stereotaxic frame (David Kopf Instrument, Tujunga, CA, USA) with an incision bar and ear bars for mounting support. The scalp was shaved and sterilised with 70% ethanol and betadine to prepare the incision site. Using sterile tools, a clean midline incision was created by pulling the skin back to reveal the skull. A 5 mm diameter craniotomy was performed using a handheld trephine drill. There was no disruption to the dura. The left hemisphere was craniotomized between the lambda and the bregma, 1 mm laterally to the midline, and 3 mm anterior to the bregma. The skull disk was gently removed without causing any damage to the brain tissue underneath, and any excess blood was cleaned using cotton swabs. A 4 mm diameter magnetic control pinpoint contusion impactor (PC1300; Hatteras Instrument, Cary, NC, USA) was used to cause the injury through cortical impact. With a dwell duration of 120 ms and a velocity of 3 m/s, the tip was designed to pierce 2 mm deep. The sham group received a craniotomy without suffering any harm. Animals were put back in their home cages and kept in standardised environmental settings after the operation. 24 h after the operation, the animals were slaughtered, and the damaged brain tissue was taken.

### 4.4 Lysate Preparation and Immunoblotting

Isolated zebrafish brains or retinae were snap‐frozen using liquid nitrogen and stored at −80°C until processing. For lysate preparation, the brains were homogenised in commercial RIPA buffer (Catalogue no. 786–489, G‐Biosciences) supplemented with 1 mM PMSF, a protease and phosphatase inhibitor cocktail (Catalogue no. PPC1010, Roche), and 1 μM DTT. The samples were incubated on ice for 30 min and then centrifuged at 14,000 rpm for 15 min at 4°C. The supernatant was carefully collected and stored at −80°C. For immunoblotting, lysates were mixed with Laemmli buffer to 1×, heated to 95°C for 10 min, and loaded onto an 8%–10% SDS‐PAGE gel. The gel was run at 90 V until the loading dye front exited the gel, followed by transfer to a nitrocellulose membrane at 90 V for 90 min. Membranes were blocked in 5% BSA for 1 h at room temperature, then incubated with primary antibody overnight at 4°C, followed by secondary antibody incubation at room temperature for 1 h. Immunoreactive bands were developed using SuperSignal West Pico PLUS Chemiluminescent Substrate (Catalogue no. 34580, Thermo Scientific) and imaged with a GE LAS 500 imager.

### 4.5 RNA Extraction, cDNA Synthesis and qPCR

Freshly harvested zebrafish brains or retinae were promptly rinsed in ice‐cold 1× PBS to remove debris and contaminants, then snap‐frozen in liquid nitrogen and stored at −80°C for later processing. A pool of five brains was combined for each time point to create one biological replicate, with three biological replicates collected (*n* = 3 per timepoint).

RNA was extracted using the RNeasy Lipid Tissue Mini Kit (Catalogue no. 74804, QIAGEN) following the manufacturer's instructions. In brief, brain tissues were homogenised in QIAzol Lysis Reagent with a mechanical homogeniser. After homogenization, chloroform was added to the lysate, followed by centrifugation at 12,000 g for 15 min at 4°C to separate the phases. The upper aqueous phase, containing RNA, was carefully transferred to a new tube. Ethanol was added to this phase to precipitate the RNA, which was then bound to the kit's spin column. On‐column DNase treatment was performed using the RNase‐Free DNase (Catalogue no. 79254, Qiagen) to ensure the RNA was free of DNA. The RNA was washed using the kit's wash buffers to remove contaminants and then eluted in RNase‐free water. The RNA's quality and quantity were evaluated using a NanoDrop UV spectrophotometer.

According to the manufacturer's instructions, cDNA was synthesised from 1 μg of total RNA using the PrimeScript 1st Strand cDNA Synthesis Kit (Catalogue no. 6110A, Takara). Following cDNA synthesis, quantitative PCR (qPCR) was performed using iTaq Universal SYBR Green Supermix (Catalogue no. 1725124, BioRad) on a BioRad CFX96 Real‐Time PCR machine. The *rpl13a* gene served as the internal control for normalising gene expression data. All qPCR reactions were conducted in triplicate to ensure reliability and reproducibility. The amplification protocol included an initial denaturation step, followed by amplification at the appropriate annealing temperature and a final melt curve analysis to confirm product specificity. Relative gene expression was calculated using the ΔΔCt method, with fold changes in gene expression determined compared to the control group. The primer sequences are included in Table S1.

### 4.6 Cryoblock Preparation and Sectioning

Freshly harvested retina and brain samples were fixed in 4% PFA overnight at 4°C. The samples were processed through a sucrose gradient with rotation, embedded in OCT medium, and cryosectioned at 8 μm thickness using a Leica Cryostat. Sections were mounted on slides, air‐dried overnight, and stored at 4°C until immunofluorescence staining.

### 4.7 Paraffin Block Preparation and Sectioning

Brain tissue was fixed in 4% PFA for 2 h at 4°C, followed by dehydration in an ethanol gradient and clearing with xylene. The tissue was then embedded in paraffin, and blocks were stored at 4°C. Sections of 5 μm thickness were obtained using a Medimeas microtome, mounted on slides, and air‐dried overnight. These slides were stored at 4°C for Toluidine Blue O staining or immunofluorescence staining.

### 4.8 Histological Assessment

Paraffin‐embedded sections heated at 60°C for 10 min. These were then dewaxed in xylene and rehydrated through a graded ethanol series (100%, 95%, 80%, and 70%) before being rinsed in 1× PBS. For histological staining, sections were incubated in 1% Toluidine Blue O for 30–45 s, then dried on a hot plate and mounted using a DPX mounting medium. The prepared slides were examined under a brightfield microscope to assess histological features and cellular architecture.

### 4.9 BrdU/EdU Labeling and Immunofluorescence Staining

Zebrafish were incubated in a 5 mM BrdU/EdU solution for 4 h prior to sacrifice to label proliferating cells. For immunofluorescence, sections were first dewaxed by incubating the slides at 60°C, followed by overnight treatment with xylene to remove paraffin. After dewaxing, the slides were rehydrated through a graded ethanol series (100%, 95%, 80%, and 70%) and then washed thoroughly in 1× PBS. Antigen retrieval was performed by boiling the slides in 10 mM sodium citrate buffer (pH 6.0) for 10 min, then cooling them to room temperature. To minimize nonspecific binding, the slides were blocked with 1× normal goat serum for 1 h at room temperature. Primary antibodies were incubated overnight at 4°C in a humidified chamber, and after washing in 1× PBS, secondary antibodies were incubated at room temperature for 1 h. DAPI (1 μg/mL) was used to counterstain the nuclei for 5 min, followed by a brief PBS wash. Slides were mounted using a Mowiol mounting medium to preserve fluorescence. Retinal sections underwent additional treatment with 2 N HCl at 37°C for 20 min, followed by neutralization with 100 mM sodium borate (pH 8.5) twice, before standard immunofluorescence procedures [83]. Imaging was done using a Leica SP8 confocal microscope at 10× and 63× magnification. High‐resolution images were acquired and analyzed for detailed cellular and subcellular localization of the targets of interest.

Primary antibodies used in the study: anti‐HCFC1 (catalogue no. A301‐399A, Bethyl laboratories), anti‐OGT (catalogue no. sc 74,546, Santa Cruz), anti‐RL2 (catalogue no. ab2739, Abcam), anti‐β‐actin (catalogue no. sc47778, Santa Cruz), anti‐Gapdh (catalogue no. SAB2701826, Sigma‐Aldrich), anti‐GFAP (catalogue no. ab154474, Abcam), anti‐BrdU (catalogue no. DSHB‐S1‐862, DSHB), anti‐PCNA (catalogue no. sc25280, Santa Cruz), anti‐HuC/D (catalogue no. A21271, Invitrogen), anti‐NeuroD1 (catalogue no. ab60704, Abcam), anti‐YAP (catalogue no. sc10119, Santa Cruz), anti‐YAP (catalogue no. sc‐365,644, Santa Cruz), anti‐Lin28a (catalogue no. a177, Cell signalling Technology), anti‐S100β (catalogue no. ab41548, Abcam), anti‐Glutamine synthetase (catalogue no. MAB302, Sigma Aldrich), and EdU (Click‐iT EdU, catalogue no. C10337, Thermofischer).

### 4.10 Morpholino Injections and Imaging

The zebrafish embryos were injected with an appropriate concentration of lissamine‐tagged morpholino at a single‐cell stage using a microinjector. The morpholino sequences were as follows: *hcfc1a* mRNA splicing (5′‐ACCATTAAAGAACTTTCTTACCTGT‐3′), *hcfc1b* translation blocking (5′‐ACCGAAGTGCCTGAAGAAGCCATGT‐3′), *yap1* translation blocking (5′‐CTCTTCTTTCTATCCAACAGAAACC‐3′) [51, 65]. The control morpholino (5′‐CCTCTTACCTCAGTTACAATTTATA‐3′) was standard offered by GeneTools Inc., USA. The survival of embryos was calculated at 24 hpf and live imaging of embryos was performed in methylcellulose medium via Zeiss Stemi DV6 microscope or Nikon SMZ18 microscope. In adult zebrafish, morpholinos were injected alongside retinal injury into the vitreous at a concentration of 0.5 mM using a Hamilton syringe of 2 μL volume capacity. Morpholino delivery to retinal cells was accomplished by electroporation, as previously described (Fausett et al., 2008).

### 4.11 mRNA Transfection

*yap5SA* gene clone was linearized and synthesis of capped mRNA was done using the mMESSAGE mMACHINE SP6 (AM1340, Thermo Fisher Scientific) in vitro transcription system. The *yap5SA* mRNA transfection was performed by preparing a transfection mixture containing equal volumes of two solutions, i.e., 4–5 μg of mRNA mixed with HBSS and Lipofectamine MessengerMax reagent (catalogue no. LMRNA001; Invitrogen) mixed with HBSS. The solutions were kept at room temperature for 10 min, then mixed dropwise and incubated at room temperature for 30 min. This mixture was used for injection in zebrafish retina and electroporated as described previously [23, 84].

### 4.12 Inhibitor Treatment

Verteporfin (catalogue no. SML0534, Sigma Aldrich) and OSMI‐1 (catalogue no. SML1621, Sigma Aldrich) were dissolved in DMSO to a final concentration of 5 mg/mL. The drugs were diluted in system water to final working concentrations of 5 μM for verteporfin and 50 μM for OSMI‐1. Zebrafish were immersed in the solution, with the solution replaced every alternate day for OSMI‐1 and daily for verteporfin. Zebrafish in system water containing the corresponding DMSO concentration served as controls.

### 4.13 Cell Culture

SH‐SY5Y cells were cultured in DMEM F12 supplemented with 10% FBS and 1% Penicillin–Streptomycin, maintained at 37°C and 5% CO2. Experiments were performed in triplicate, with cells seeded at a density of 2.8–4 × 10^4^ cells/cm^2^. For verteporfin treatment, the media was supplemented with 4 μM verteporfin, and the treatment was done for 24 h. Cells treated with the corresponding amount of DMSO supplemented media were used as a control.

### 4.14 Co‐Immunoprecipitation

For the Co‐IP assay, 20 retinae were pooled for each sample in lysis buffer supplemented with PMSF and protease inhibitor cocktail and stored at −80°C. The samples were thawed in water, followed by pipetting and vortexing to obtain retinal lysate. The lysates were mixed with fresh lysis buffer supplemented with PMSF and protease inhibitor cocktail and centrifuged at 10000 g for 10 min at 4°C. The supernatant was subjected to pulldown using an anti‐HCFC1 antibody. The rabbit IgG was used as a control in the experiment. The samples were boiled in Laemmli buffer and run on 8% SDS‐PAGE gel. The individual lanes were cut and used for mass‐spectrometry analysis. The gel pieces (whole lane) were in‐gel trypsin digested and subjected to Triple‐TOF LC–MS.

### 4.15 Statistical Analysis

All experiments were conducted in biological triplicates (with each replicate consisting of pooled samples for qRT‐PCR experiments). Data are presented as mean ± standard error of the mean (SEM). A Student's *t*‐test (two‐tailed) was applied for comparisons between two groups, with statistical significance determined by a *p*‐value of less than 0.05.

## Author Contributions

P.P.S., S.B., P.S., R.R., and S.M. conceived the study and designed the experiments. Brain regeneration experiments were done by P.P.S. and S.B. Retinal regeneration experiments were done by P.S., O.M.D., and K.Y. Rat TBI experiments were done by A., R.C., and S.P. All authors contributed to the analysis, data discussion, and the creation of the manuscript's final draft.

## Conflicts of Interest

The authors declare no conflicts of interest.

## Supporting information


**Figure S1:** Validation of telencephalic stab‐wound injury model: (A) Telencephalic stab wound‐injury model in Zebrafish brain wherein the right hemisphere is injured with a 30G needle and the left uninjured hemisphere acts as contralateral control. (B) Histological analysis of regenerating brains (1, 4, 7 and 30 dpl) using Toluidine Blue O stain. Scale bar is 100 μm. (C) Immunoblot of uninjured control and regenerating brains with anti‐PCNA (proliferation marker) and anti‐β‐actin as a loading control. (D) Immunostaining of regenerating 4 dpl brain with anti‐BrdU (red) showing co‐localization with DAPI (blue). Scale bar is 100 μm with higher magnification (scale bar 50 μm) in the lower right corner of the merged panel. OB‐ Olfactory bulb, Tel‐ Telencephalon, TeO‐Optic tectum, CC‐Cerebellum.
**Figure S2:** Hcfc1 expression in Zebrafish brain: (A) mRNA expression levels of *hcfc1a* and *hcfc1b* in zebrafish brains (*n* = 3). Significance is represented as n.s. for non‐significant, * for *p*‐value < 0.05, ** for *p*‐value < 0.01 and *** for *p*‐value < 0.001. (B) Immunostaining of control Zebrafish brain telencephalon with anti‐HCFC1 (green). Counterstaining is done with DAPI (blue). Scale bar is 100 μm.
**Figure S3:** *O*‐GlcNAc and OGT expression during zebrafish brain regeneration: (A) Immunostaining of regenerating brains at 7 dpl with anti‐HCFC1(green) and anti‐OGT (red). Merged panel (A3 and A6) show co‐localization of OGT expressing cells with HCFC1 expressing cells. Scale bar for panels A1‐A3 is 100 μm and for panels A4‐A6 is 50 μm. (B) Immunoblot of regenerating brains (uninjured control, 4, 7, 14 and 30 dpl) with anti‐RL2 (*O*‐GlcNAc). Anti‐β‐actin was used as a loading control. (C) Quantification of *O*‐GlcNAc levels during regeneration is shown as a bar graph (*n* = 3) Significance is represented as n.s. for *p*‐value > 0.05 and * for *p*‐value < 0.05.
**Figure S4:** Hcfc1 is upregulated in radial glial progenitor cells during brain regeneration. (A) Immunostaining of zebrafish telencephalon at 7 days post‐lesion (dpl; A1–A5) and 14 dpl (A6–A10) with anti‐HCFC1 (green) and anti‐GFAP (red). A1–A2 and A6–A7 show low‐magnification views; boxed regions are shown at higher magnification in A3–A5 and A8–A10, respectively. Merged panels include nuclear stain DAPI (blue). Scale bars: A1–A2, A6–A7 = 100 μm; A3–A5, A8–A10 = 50 μm. (B) Immunostaining at 7 dpl with anti‐HCFC1 (green) and anti–glutamine synthetase (GS, red). Low‐magnification merged image is shown in B1‐B4; boxed region is shown at higher magnification in B5–B8. Merged panels include DAPI (blue). Scale bars: B1–B4 = 50 μm; B5–B8 = 20 μm. GS, glutamine synthetase.
**Figure S5:** Traumatic brain injury in Rat model. (A) qRT‐PCR of *il1b, tnfa and cox2* in control (sham) and injured brain at 1 and 7 dpi. (B) qRT‐PCR of *hcfc1* and *ogt* in control (sham) and injured brain at 1 and 7 dpl. β‐actin was used as housekeeping gene (*n* = 3). Significance is represented as n.s. for non‐significant, * for *p*‐value < 0.05, ** for *p*‐value < 0.01 and *** for *p*‐value < 0.001. (C) Immunoblot of Control (sham) and injured rat brain at 7 dpl with anti‐HCFC1 and anti‐PCNA. β‐actin was used as a loading control.
**Figure S6:** Hcfc1 interacting proteins during retinal regeneration. (A) The Hcfc1 interacting proteins during retinal regeneration at 2 dpi, based on literature search, are known to be involved in various essential processes in regeneration. (B) Gene ontology enrichment analysis of Hcfc1 interacting proteins in control retina. (B) Gene ontology enrichment analysis of Hcfc1 interacting proteins in regenerating retina at 2 dpi.
**Figure S7:** Effects of OGT activity inhibition on *O*‐GlcNAcylation levels and apoptosis during zebrafish brain regeneration. (A) Immunostaining of regenerating 7 dpl brain (DMSO control) and OSMI1 (50 μM) treated brains with anti‐HCFC1 (green, white arrow) and anti‐*O*‐GlcNAc (glycosylation marker (red), white arrow). Scale bar is 100 μm. (B) Assessment of cell death via TUNEL assay in 7 dpl regenerating (DMSO control) and 7 dpl OSMI1 (50 μM) treated brain. Scale bar is 100 μm. (C) Quantification of no. of TUNEL‐positive cells compared between the uninjured and injured telencephalic hemispheres of DMSO control and OSMI‐1 treated (50 μM) 7 dpl brains (*n* = 3). Significance is represented as n.s. for non‐significant, * for *p*‐value < 0.05, ** for *p*‐value < 0.01 and *** for *p*‐value < 0.001. (D) Immunoblot of regenerating control brain (DMSO treated) and regenerating OSMI‐1 treated (50 μM) brain at 7 dpl with anti‐Phosphorylated YAP. (E) Immunoblot of regenerating control brain (DMSO treated) and regenerating OSMI‐1 treated (50 μM) brain at 7 dpl with anti‐Total YAP. Anti‐β‐actin was used as loading control for both the immunoblots.
**Figure S8:** YAP is upregulated in progenitor, neuronal and proliferating cells during zebrafish brain regeneration. (A) Immunostaining of regenerating telencephalon at 4 days post‐lesion (dpl) with anti‐YAP (green) and anti–glutamine synthetase (GS, red). DAPI (blue) marks nuclei. A1 shows low‐magnification view; boxed region is shown at higher magnification in A2–A5. Merged panels (A1, A5) highlight co‐localization of DAPI, YAP and GS (yellow arrows). Scale bars: A1 = 50 μm; A2–A5 = 20 μm. (B) Immunostaining at 4 dpl with anti‐YAP (green) and anti‐HuC/D (red). DAPI in blue. B1 shows low‐magnification view; boxed region is shown at higher magnification in B2–B5. Merged panels (B1, B5) highlight DAPI, YAP and HuC/D co‐localization (yellow arrows). Scale bars: B1 = 50 μm; B2–B5 = 20 μm. (C) Immunostaining at 7 dpl with anti‐YAP (red) and anti–S100β (green), with DAPI in blue. C1–C4 show merged views with co‐localization indicated by yellow arrows. Scale bar: 50 μm. (D) Immunostaining at 4 dpl with anti‐YAP (green) and anti‐BrdU (red). DAPI in blue. D1–D4 show merged views with co‐localization indicated by yellow arrows. Scale bar: 50 μm. GS, Glutamine synthetase.
**Figure S9:** Yap regulates Hcfc1 levels. (A) qRT‐PCR of regenerating retina at 2 dpi injected with control morpholino and morpholino against *yap1* (0.5 mM and 1 mM). (B) Immunoblot of control (DMSO treated) and verteporfin (4 μM) treated SH‐SY5Y cells with anti‐HCFC1. Anti‐β‐actin was used as a loading control.
**Table S1:** List of Primers used in the study.
**Table S2:** List of Hcfc1 interacting proteins in zebrafish control retina (0 dpi).
**Table S3:** List of Hcfc1 interacting proteins in zebrafish regenerating retina at 2 dpi.

## Data Availability

All data generated during this study is included in this published article and its Supporting Information files. Mass‐spectrometry data has been deposited at the MassIVE repository (https://massive.ucsd.edu/) as MSV000097697 (ftp://msv000097697@massive‐ftp.ucsd.edu).
