# Supplementary material for: Hcfc1 and Ogt Mediate Zebrafish CNS Regeneration Through Hippo/Yap Signalling
Source: Cell Prolif. 2025 Oct 17;59(5):e70132. doi: 10.1111/cpr.70132 (PMC13114781; doi:10.1111/cpr.70132)

**Supporting information**

**Hcfc1 and Ogt mediate zebrafish CNS regeneration through Hippo/Yap signaling**

*Priyanka P. Srivastava*$\dagger^{a}$*, Sidharth Bhasin*$\dagger^{a,b}$*, Poonam Sharma*$\dagger^{c}$*, Omkar Mahadeo Desai^c,d^, Kshitiz Yadav^c^, Ayushma^a^, Rohan Chakraborty^e^, Suhel Parvez^e^, Rajesh Ramachandra*$n^{*c}$*, Shilpi Minoch*$a^{*a}$

*^a^ Kusuma School of Biological Sciences, Indian Institute of Technology Delhi (IITD), Hauz Khas, New Delhi 110016, India*

*^b^ Center for Regenerative Therapies Dresden (CRTD), Technische Universität Dresden, Fetscherstr. 105, 01307 Dresden, Germany*

*^c^ Room No. 3F10, AB-1, Department of Biological Sciences, IISER Campus, Knowledge City, Sector 81, SAS Nagar (Mohali), P.O Mohali 140306, India.*

*^d^ Model Systems for Infection and Immunity (MSYS) Helmholtz Centre for Infection*

*Research, Inhoffenstr. 7, 38124 Braunschweig, Germany.*

*^e^ Department of Medical Elementology and Toxicology, Jamia Hamdard University, New Delhi 110062, India*

* Co-corresponding Authors: [rajeshra@iisermohali.ac.in](mailto:rajeshra@iisermohali.ac.in) and [sminocha@bioschool.iitd.ac.in](mailto:sminocha@bioschool.iitd.ac.in)

† Authors contributed equally


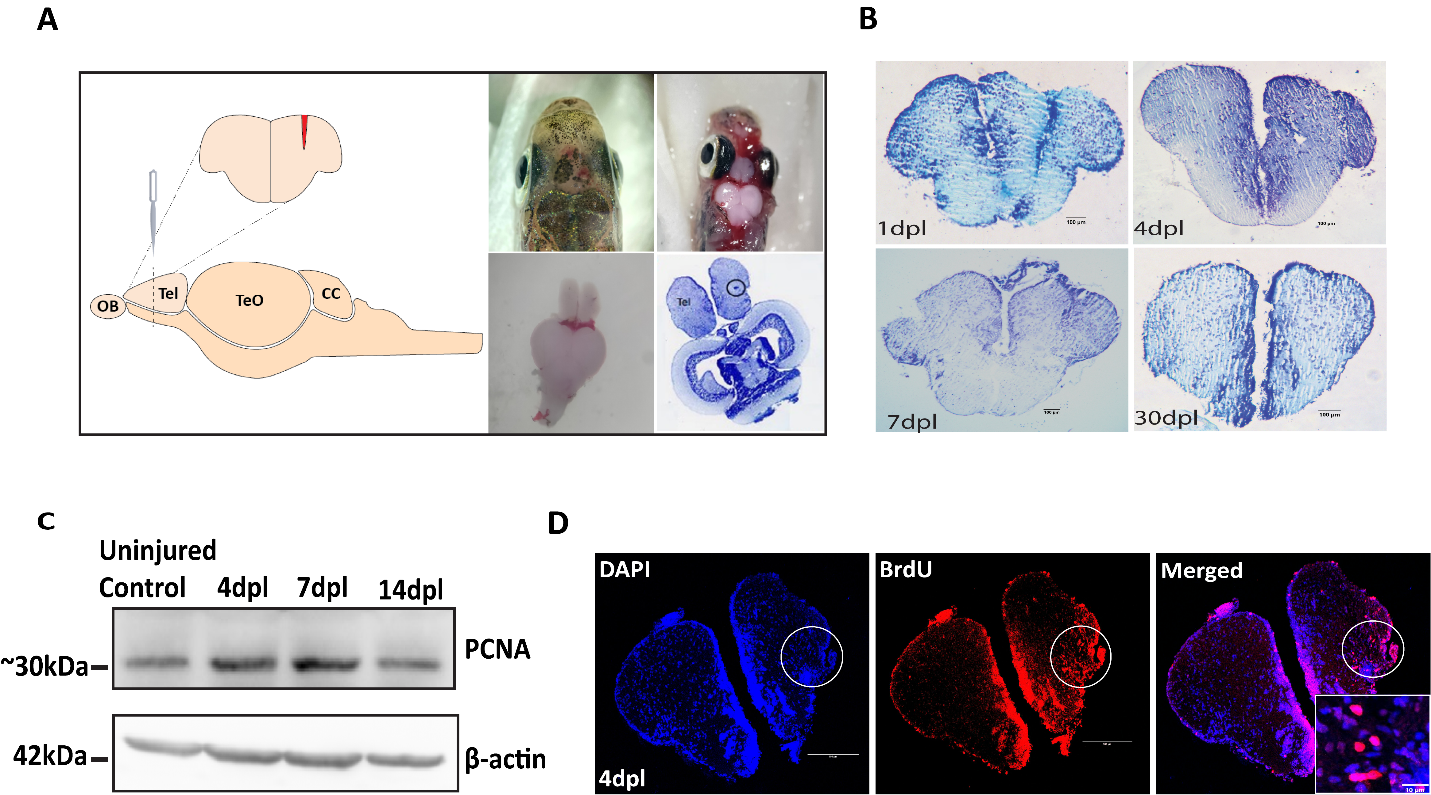


**Figure S1 Validation of telencephalic stab-wound injury model**: (A) Telencephalic stab wound-injury model in Zebrafish brain wherein the right hemisphere is injured with a 30G needle and the left uninjured hemisphere acts as contralateral control. (B) Histological analysis of regenerating brains (1 dpl, 4 dpl, 7 dpl and 30 dpl) using Toluidine Blue O stain. Scale bar is 100μm. (C) Immunoblot of uninjured control and regenerating brains with anti-PCNA (proliferation marker) and anti-β-actin as a loading control. (D) Immunostaining of regenerating 4dpl brain with anti-BrdU (red) showing co-localization with DAPI (blue). Scale bar is 100μm with higher magnification (scale bar 50μm) in the lower right corner of the merged panel). OB- Olfactory bulb, Tel- Telencephalon, TeO-Optic tectum, CC-Cerebellum


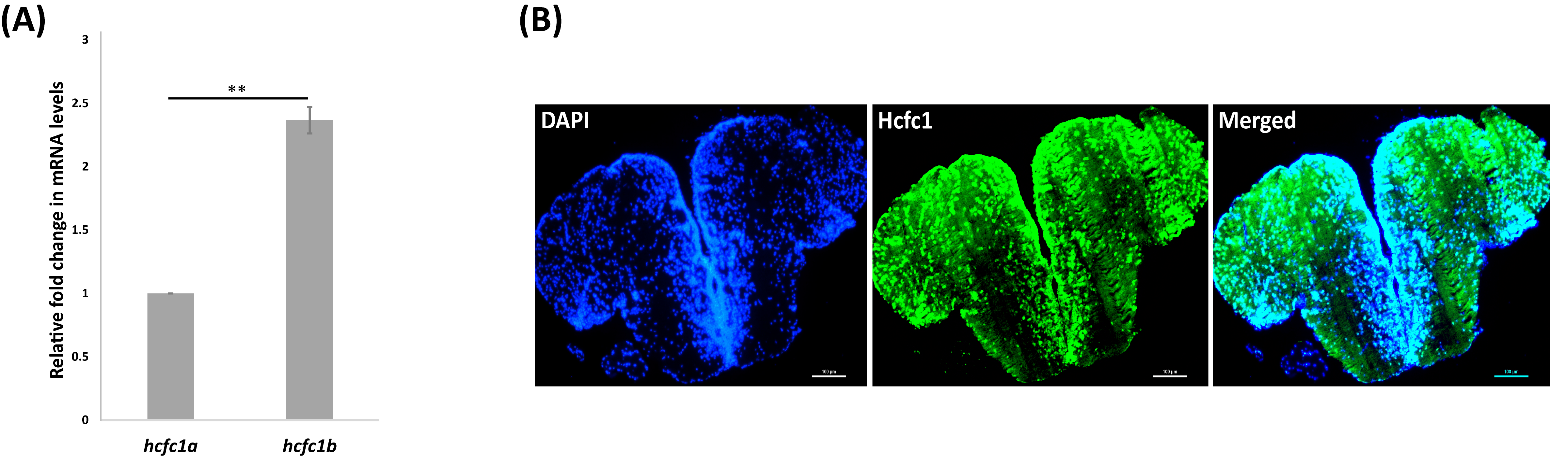


**Figure S2 Hcfc1 expression in Zebrafish brain**: (A) mRNA expression levels of *hcfc1a* and *hcfc1b* in zebrafish brains (n=3). Significance is represented as n.s. for non-significant, * for P-value<0.05, ** for p-value<0.01 and *** for p-value<0.001. (B) Immunostaining of control Zebrafish brain telencephalon with anti-HCFC1 (green). Counterstaining is done with DAPI (blue). Scale bar is 100μm.


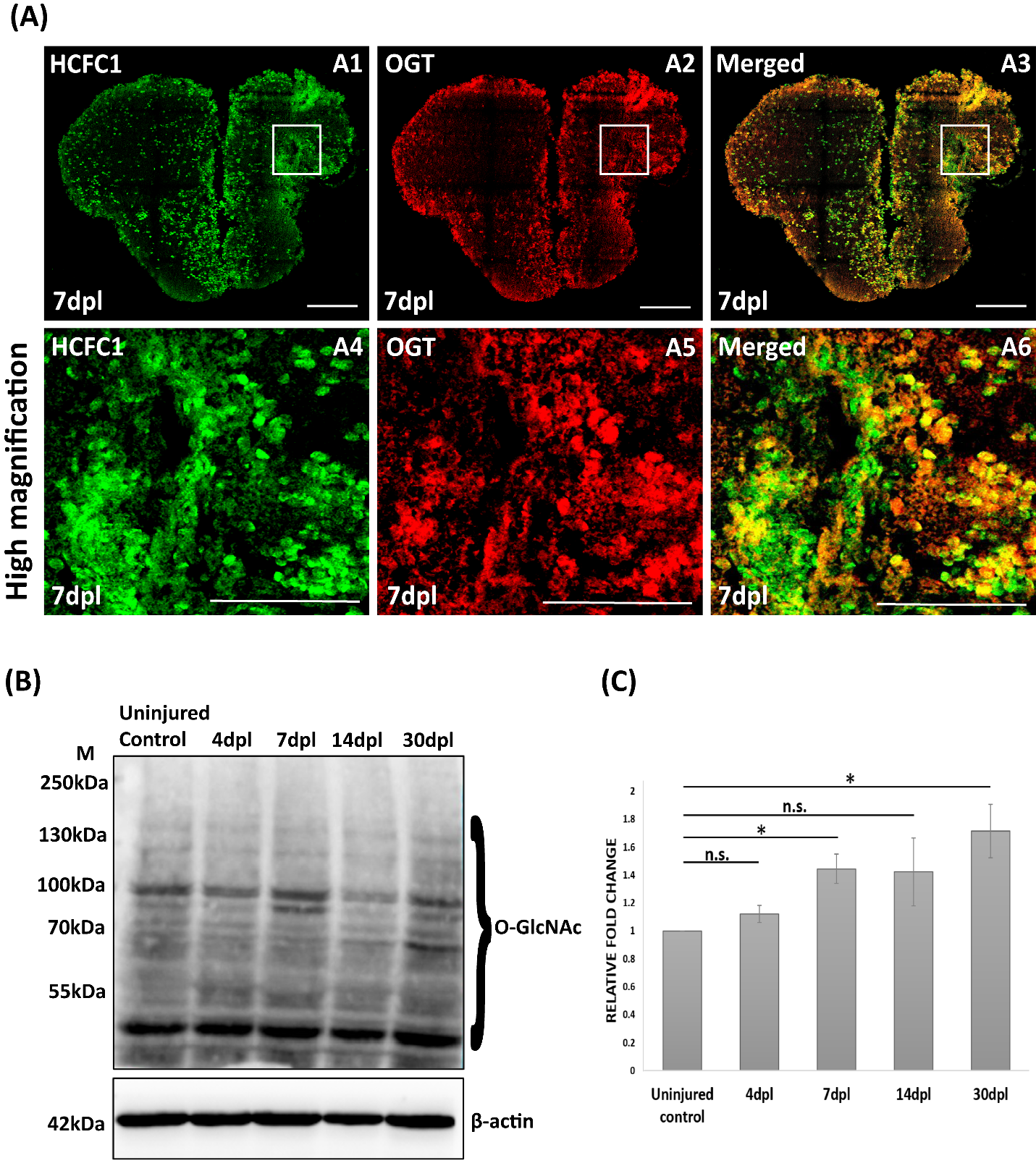


**Figure S3: O-GlcNAc and OGT expression during zebrafish brain regeneration**: (A) Immunostaining of regenerating brains at 7 dpl with anti-HCFC1(green) and anti-OGT (red). Merged panel (A3 and A6) show co-localization of OGT expressing cells with HCFC1 expressing cells. Scale bar for panels A1-A3 is 100μm and for panels A4-A6 is 50μm. (B) Immunoblot of regenerating brains (uninjured control, 4dpl, 7dpl, 14dpl and 30dpl) with anti-RL2 (O-GlcNAc). Anti-β-actin was used as a loading control. (C) Quantification of O-GlcNAc levels during regeneration is shown as a bar graph (n=3) Significance is represented as n.s. for p-value>0.05 and * for P-value<0.05.


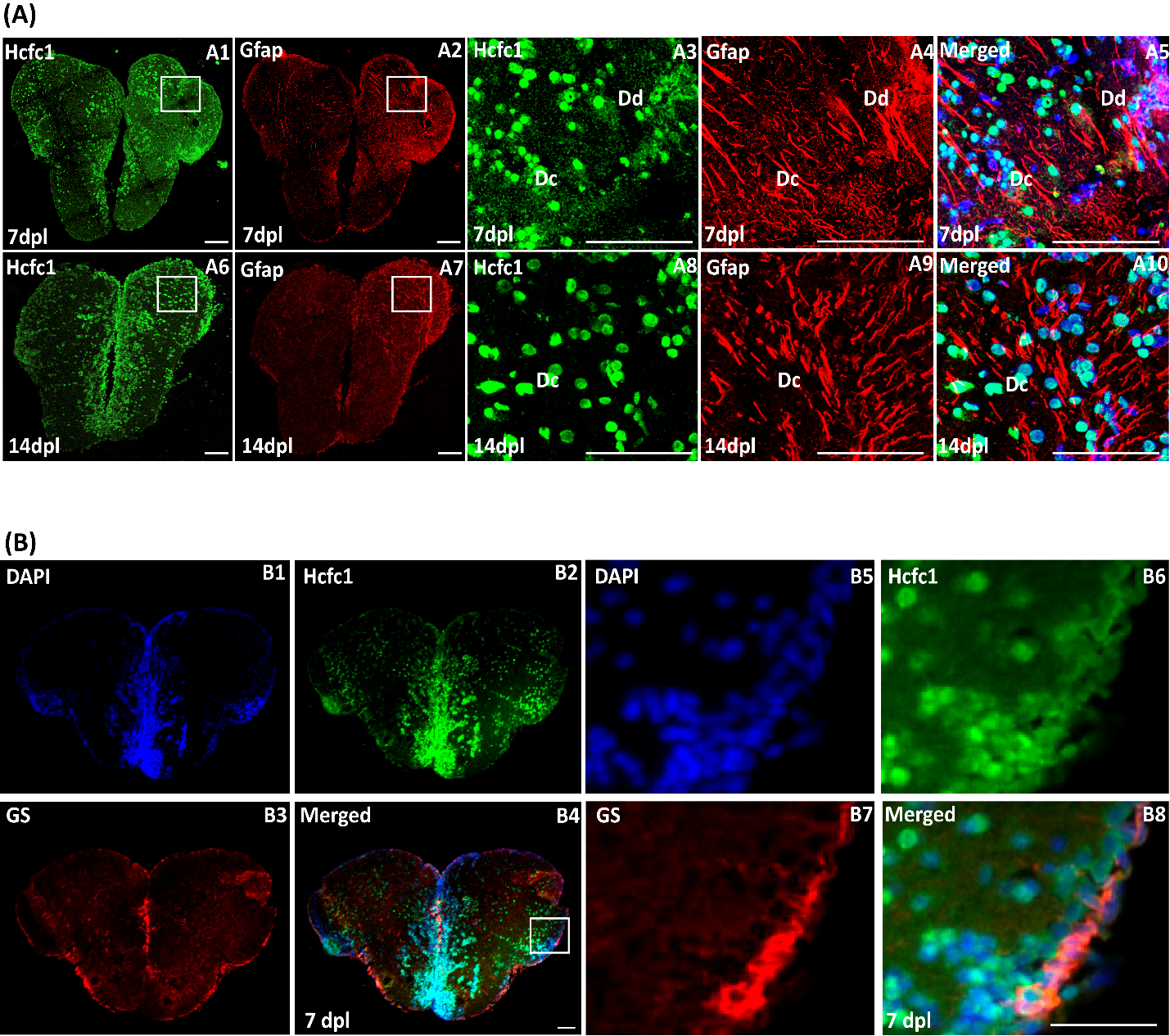


**Figure S4. Hcfc1 is upregulated in radial glial progenitor cells during brain regeneration.** (A) Immunostaining of zebrafish telencephalon at 7 days post-lesion (dpl; A1–A5) and 14 dpl (A6–A10) with anti-HCFC1 (green) and anti-GFAP (red). A1–A2 and A6–A7 show low-magnification views; boxed regions are shown at higher magnification in A3–A5 and A8–A10, respectively. Merged panels include nuclear stain DAPI (blue). Scale bars: A1–A2, A6–A7 = 100 μm; A3–A5, A8–A10 = 50 μm. (B) Immunostaining at 7 dpl with anti-HCFC1 (green) and anti–glutamine synthetase (GS, red). Low-magnification merged image is shown in B1-B4; boxed region is shown at higher magnification in B5–B8. Merged panels include DAPI (blue). Scale bars: B1–B4 = 50 μm; B5–B8 = 20 μm. GS, glutamine synthetase.


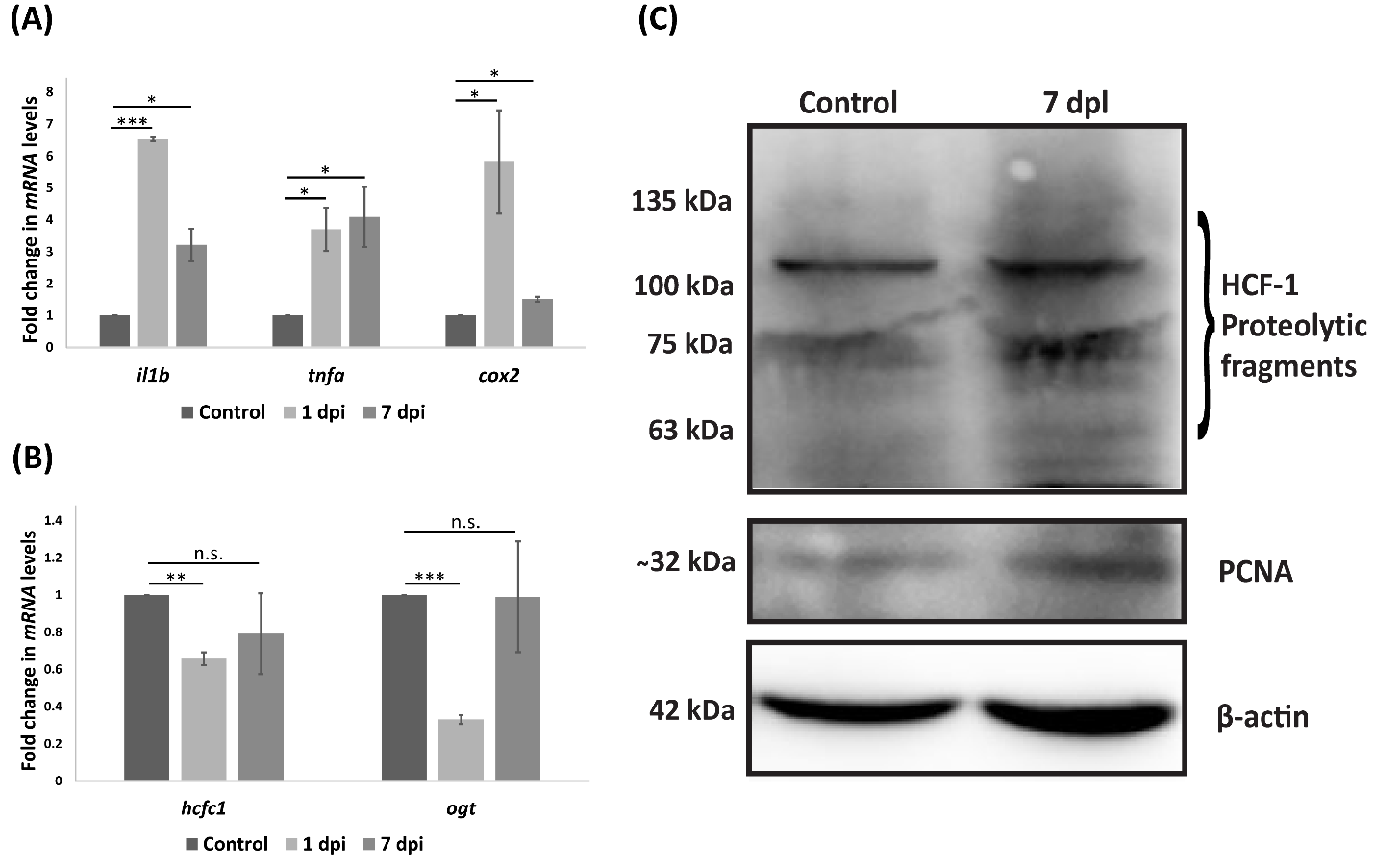


**Figure S5: Traumatic brain injury in Rat model.** (A) qRT-PCR of *il1b, tnfa and cox2* in control (sham) and injured brain at 1 dpi and 7 dpi. (B) qRT-PCR of *hcfc1* and *ogt* in control (sham) and injured brain at 1 dpl and 7 dpl. β-actin was used as housekeeping gene (n=3). Significance is represented as n.s. for non-significant, * for P-value<0.05, ** for p-value<0.01 and *** for p-value<0.001. (C) Immunoblot of Control (sham) and injured rat brain at 7 dpl with anti-HCFC1 and anti-PCNA. β-actin was used as a loading control.


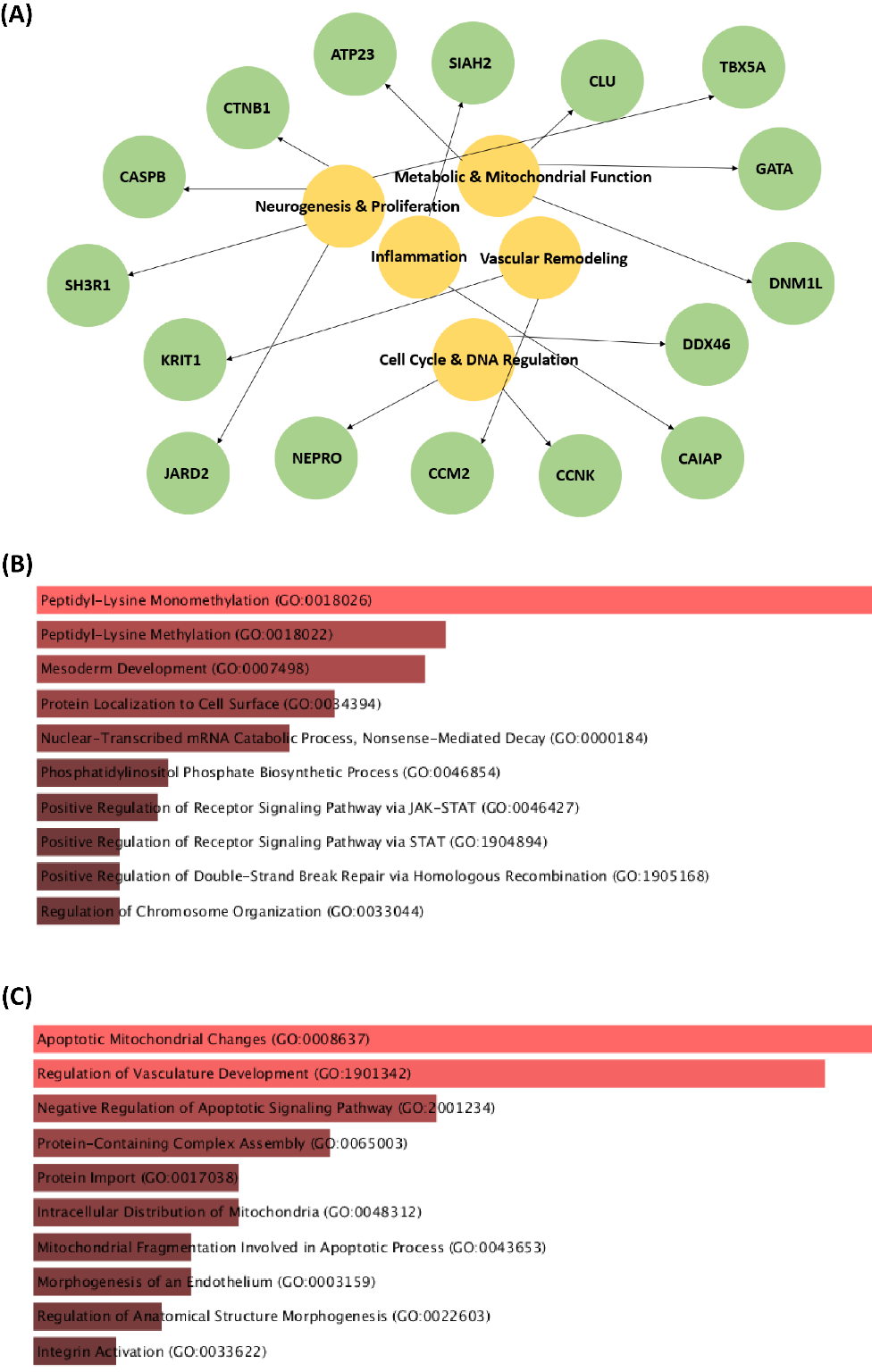


**Figure S6: Hcfc1 interacting proteins during retinal regeneration**. (A) The Hcfc1 interacting proteins during retinal regeneration at 2 dpi, based on literature search, are known to be involved in various essential processes in regeneration. (B) Gene ontology enrichment analysis of Hcfc1 interacting proteins in control retina. (B) Gene ontology enrichment analysis of Hcfc1 interacting proteins in regenerating retina at 2 dpi.


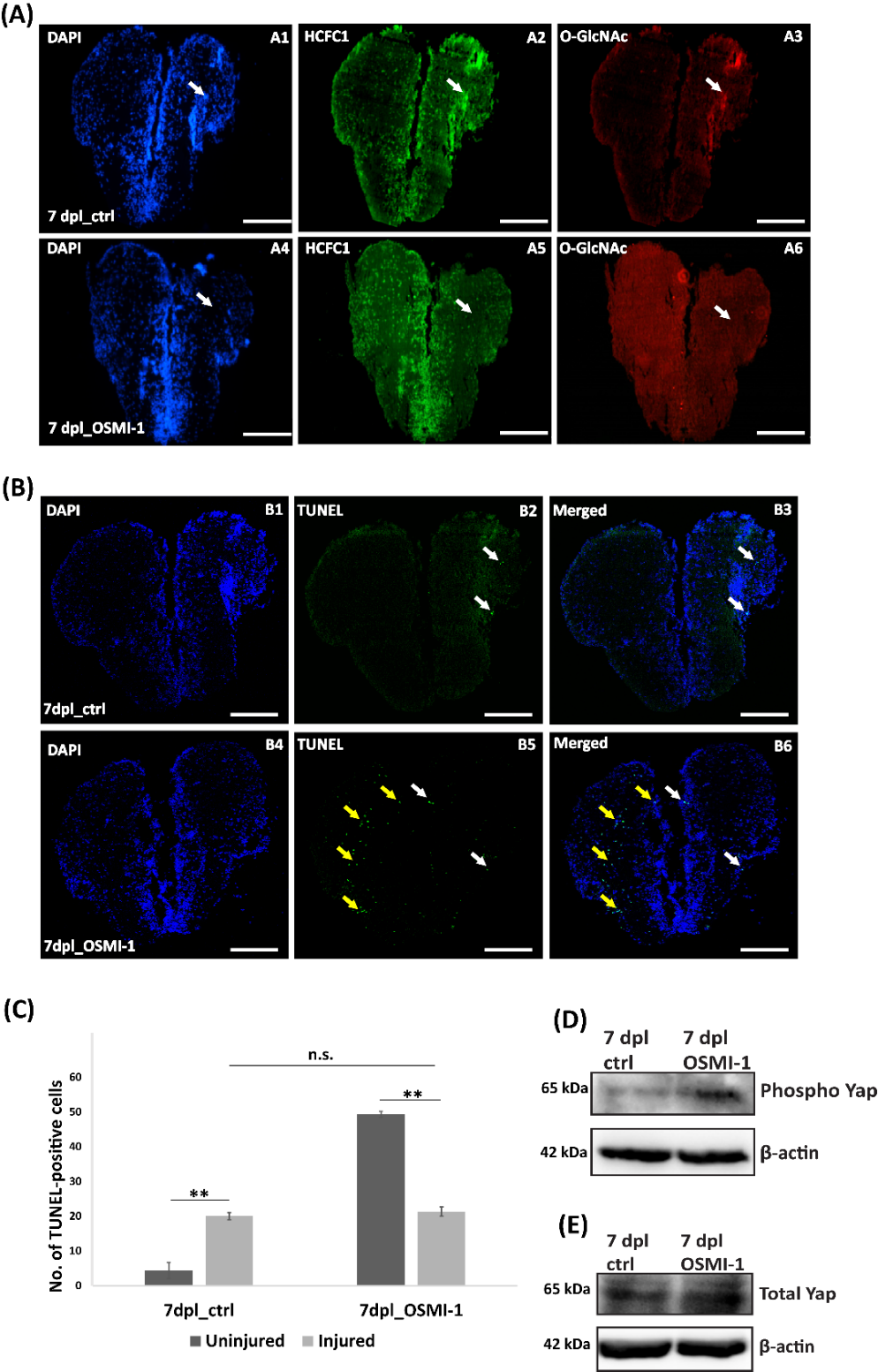


**Figure S7: Effects of OGT activity inhibition on O-GlcNAcylation levels and apoptosis during zebrafish brain regeneration.** (A) Immunostaining of regenerating 7dpl brain (DMSO control) and OSMI1 (50µM) treated brains with anti-HCFC1 (green, white arrow) and anti-O-GlcNAc (glycosylation marker (red), white arrow). Scale bar is 100μm. (B) Assessment of cell death via TUNEL assay in 7dpl regenerating (DMSO control) and 7dpl OSMI1 (50µM) treated brain. Scale bar is 100μm. (C) Quantification of no. of TUNEL-positive cells compared between the uninjured and injured telencephalic hemispheres of DMSO control and OSMI-1 treated (50µM) 7dpl brains (n=3). Significance is represented as n.s. for non-significant, * for P-value<0.05, ** for p-value<0.01 and *** for p-value<0.001. (D) Immunoblot of regenerating control brain (DMSO treated) and regenerating OSMI-1 treated (50µM) brain at 7 dpl with anti-Phosphorylated YAP. (E) Immunoblot of regenerating control brain (DMSO treated) and regenerating OSMI-1 treated (50µM) brain at 7 dpl with anti-Total YAP. Anti-β-actin was used as loading control for both the immunoblots.


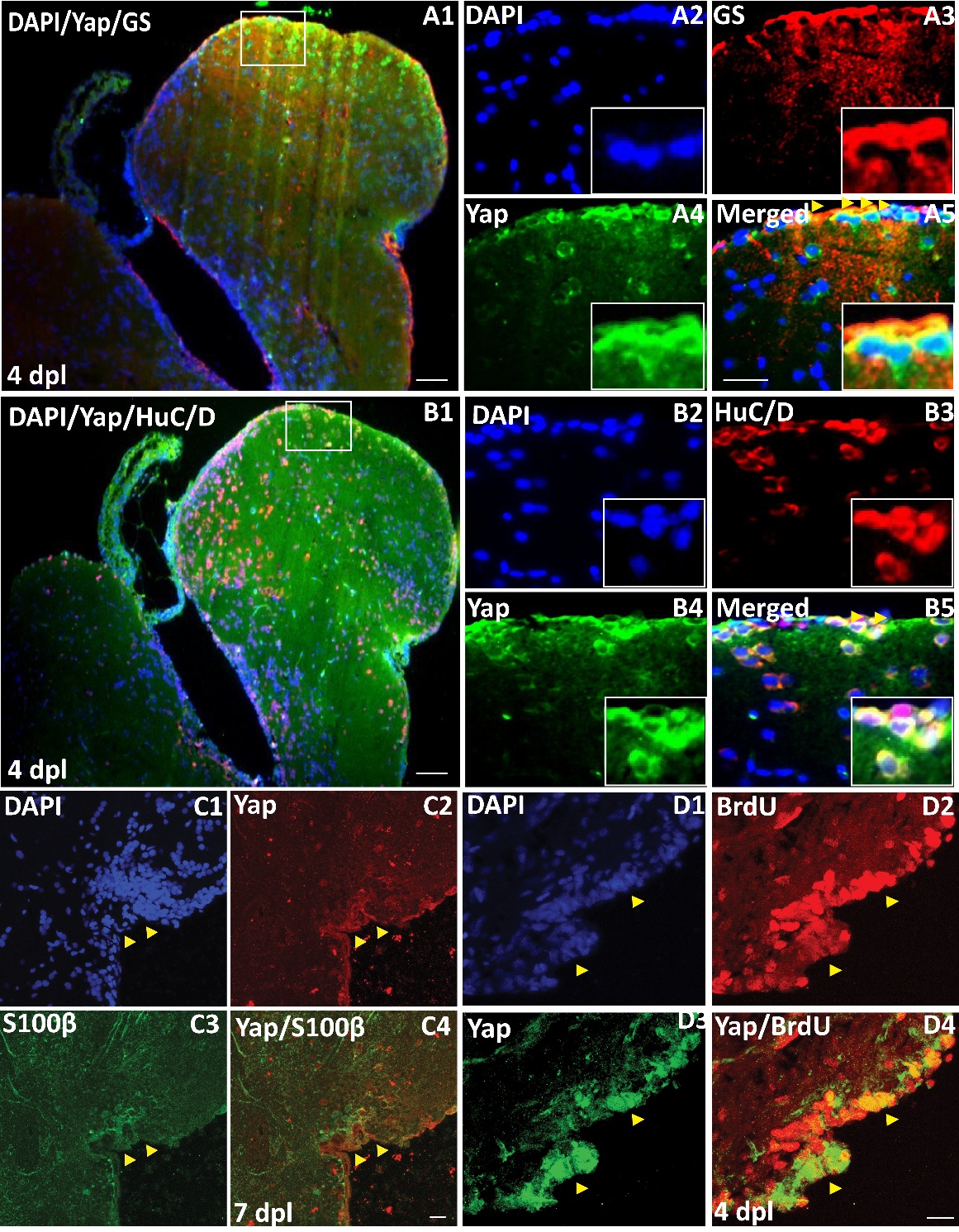


**Figure S8: YAP is upregulated in progenitor, neuronal and proliferating cells during zebrafish brain regeneration.** (A) Immunostaining of regenerating telencephalon at 4 days post-lesion (dpl) with anti-YAP (green) and anti–glutamine synthetase (GS, red). DAPI (blue) marks nuclei. A1 shows low-magnification view; boxed region is shown at higher magnification in A2–A5. Merged panels (A1, A5) highlight co-localization of DAPI, YAP and GS (yellow arrows). Scale bars: A1 = 50 μm; A2–A5 = 20 μm. (B) Immunostaining at 4 dpl with anti-YAP (green) and anti-HuC/D (red). DAPI in blue. B1 shows low-magnification view; boxed region is shown at higher magnification in B2–B5. Merged panels (B1, B5) highlight DAPI, YAP and HuC/D co-localization (yellow arrows). Scale bars: B1 = 50 μm; B2–B5 = 20 μm. (C) Immunostaining at 7 dpl with anti-YAP (red) and anti–S100β (green), with DAPI in blue. C1–C4 show merged views with co-localization indicated by yellow arrows. Scale bar: 50 μm. (D) Immunostaining at 4 dpl with anti-YAP (green) and anti-BrdU (red). DAPI in blue. D1–D4 show merged views with co-localization indicated by yellow arrows. Scale bar: 50 μm. GS, Glutamine synthetase.


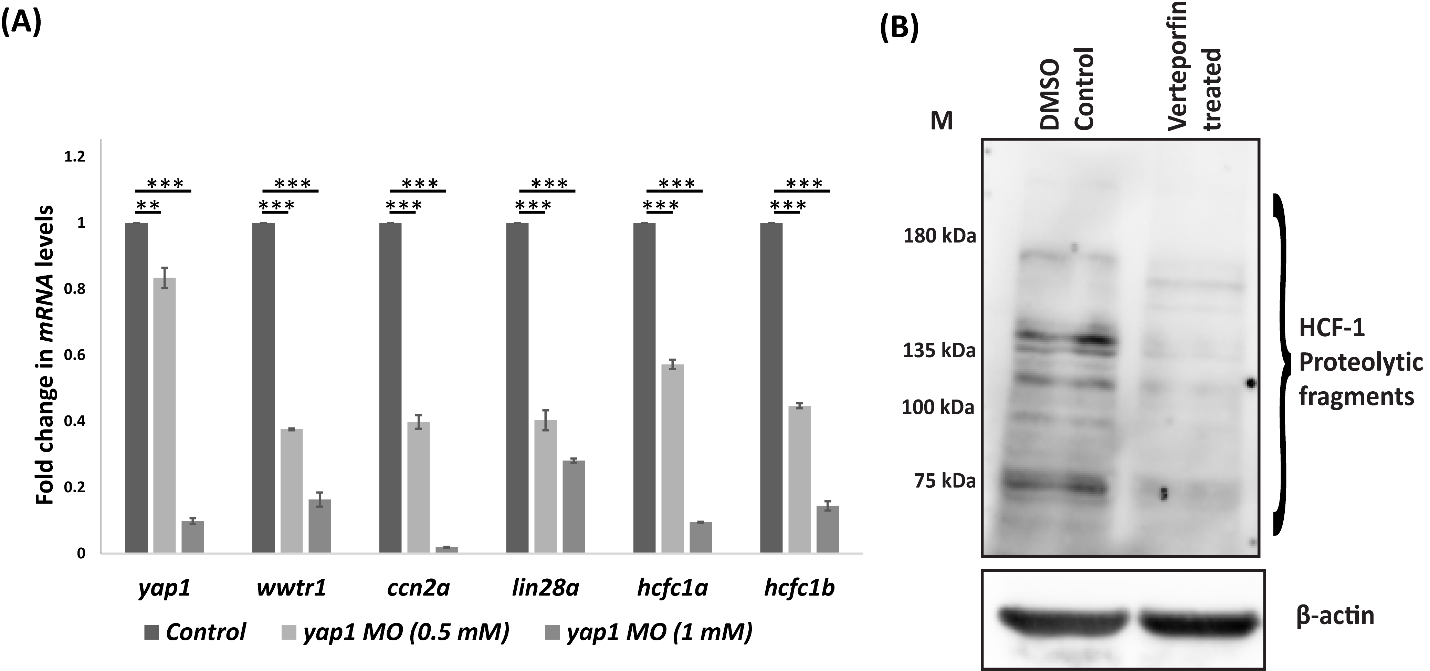


**Figure S9: Yap regulates Hcfc1 levels.** (A) qRT-PCR of regenerating retina at 2 dpi injected with control morpholino and morpholino against *yap1* (0.5 mM and 1 mM). (B) Immunoblot of control (DMSO treated) and verteporfin (4 µM) treated SH-SY5Y cells with anti-HCFC1. Anti-β-actin was used as a loading control.

Table S1: List of Primers used in the study

| **Primer name** | **Organism** | **Primer sequence** |
| --- | --- | --- |
| *hcfc1a* forward primer | Zebrafish | GCGATGGCTGCAAGAGTC |
| *hcfc1a* reverse primer | Zebrafish | GGCTAAATGCAGAGGACAGGT |
| *hcfc1b* forward primer | Zebrafish | CTACACCACCAAACCGCTA |
| *hcfc1b* reverse primer | Zebrafish | GACCAGCGCCTTTCATATCAGG |
| *stk3* forward primer | Zebrafish | GGGGAAAGGGACACCAATCT |
| *stk3* reverse primer | Zebrafish | GCAGGATAGACACCGGCTTA |
| *sav1* forward primer | Zebrafish | CTCATTGGAGCCATCCGCT |
| *sav1* reverse primer | Zebrafish | CATAACGAGGCACACTGGG |
| *neurog1* forward primer | Zebrafish | CCCAGCCCACCAATAAGGTTATCAA |
| *neurog1* reverse primer | Zebrafish | CAGGTGGTTTTCCGCAGGAG |
| *nf2b* forward primer | Zebrafish | AGTACGGGGACTACGATCCA |
| *nf2b* reverse primer | Zebrafish | TCAGCCTCGTCCCTTGTGAT |
| *mob1a* forward primer | Zebrafish | CAGACGGCACCAACATCAAG |
| *mob1a* reverse primer | Zebrafish | GGGGAAAGGGACACCAATCT |
| *amotl2a* forward primer | Zebrafish | CCCCTTCATGGTCAGATCCC |
| *amotl2a* reverse primer | Zebrafish | GCTGTGTTGGAAATAGCCTGTAA |
| *ccn2a* forward primer | Zebrafish | CTGGACGGTGCTGTAGGTTG |
| *ccn2a* reverse primer | Zebrafish | CCTCCTCTCTGTAAGCTGCTAAA |
| *frmd6* forward primer | Zebrafish | GCTGGTGTTTGTGGGTAAGAG |
| *frmd6* reverse primer | Zebrafish | TGTACAGTCGGTGGCTGTTG |
| *hbegfa* forward primer | Zebrafish | GACTGGAGAAGAACGGGCGA |
| *hbegfa* reverse primer | Zebrafish | CTCCTGCCAAACAAACACGG |
| *myca* forward primer | Zebrafish | GCAAGTGACCGGAGCAAAAA |
| *myca* reverse primer | Zebrafish | ACTCACCGGCATTTTGACACTTG |
| *gli2a* forward primer | Zebrafish | ATTGAAGCCGTCAGTCCTGG |
| *gli2a* reverse primer | Zebrafish | GCTAATCCATGAGGTCCATGC |
| *yap1* forward primer | Zebrafish | ACCCGCGTTTCGCAATGA |
| *yap1* reverse primer | Zebrafish | TCGGATGGGGTTGTTTCCAC |
| *wwtr1* forward primer | Zebrafish | GATGGAGAGAGAGCGCATCC |
| *wwtr1* reverse primer | Zebrafish | GTGATACGGCCCACTGTTGA |
| *lin28a* forward primer | Zebrafish | GGCGACCAGTCCACATTAAC |
| *lin28a* reverse primer | Zebrafish | TCTGTCTTTGTACATCCACCTGT |
| *rpl13a* forward primer | Zebrafish | TCTGGAGGACTGTAAGAGGTATGC |
| *rpl13a* reverse primer | Zebrafish | AGACGCACAATCTTGAGAGCAG |
| *il1b* forward primer | Rat | GGGATGATGACGACCTGCTAG |
| *il1b* reverse primer | Rat | TGTCGTTGCTTGTCTCCCTTG |
| *tnf* forward primer | Rat | GACCCTCACACTCAGATCATCT |
| *tnf* reverse primer | Rat | GTTGTCTTTGAGATCCATGCCA |
| *cox2* forward primer | Rat | AAAGGCCTCCATTGACCAGAG |
| *cox2* reverse primer | Rat | TCGATGTCATGGTAGAGGGC |
| *hcfc1* forward primer | Rat | CCCAAGAGCTGATGGCTGAA |
| *hcfc1* reverse primer | Rat | CAGTGCCCATGACAGCCT |
| *ogt* forward primer | Rat | GCTGCCCTTTTTCTGCTACC |
| *Ogt* reverse primer | Rat | CCGATGTGCCAACTCAGCTA |
| *b actin* forward primer | Rat | CTGTGTGGATTGGTGGCTCT |
| *b actin* reverse primer | Rat | AGCTCAGTAACAGTCCGCCT |

Table S2: List of Hcfc1 interacting proteins in zebrafish control retina (0 dpi)

| **Protein entry** | **Protein name** | **Function** |
| --- | --- | --- |
| SETD6 | N-lysine methyltransferase setd6 | Involved in modulation of histone modification, gene expression and, oxidative stress response |
| RFP4B | Rab11 family-interacting protein 4B | essential for retinal development and regulates proliferation and differentiation of retinal progenitor cells |
| SMG1 | Serine/threonine-protein kinase SMG1 | Involved in neurogenesis and neural differentiation, modulates response against oxidative stress and DNA damage in retina |
| MESD | LRP chaperone MESD | involved in retinal angiogenesis and neuroprotection, |
| DS7CB | Dehydrogenase/reductase SDR family member 7C-B | Involved in the maintenance of intracellular calcium ion homeostasis |
| ANR46 | Ankyrin repeat domain-containing protein 46 | Mediates protein-protein interaction crucial for nervous system functions |
| CR19A | Uncharacterized protein C18orf19 homolog A | Involved in mitochondrial function |
| PT117 | Protein PET117 homolog, mitochondrial | Involved in mitochondrial function and cellular energy metabolism in brain and retina |
| WDR48 | WD repeat-containing protein 48 | Regulates retinal cell maintenance and function, DNA repair and ubiquitin signaling |
| EXT1A | Exostosin-1a | Crucial for neuronal development and function |
| KPCI | Protein kinase C iota type | Regulates neuronal cell survival, differentiation and maintaining polarity, crucial for neural functioning |
| IMDH1 | Inosine-5'-monophosphate dehydrogenase 1a | Involved in maintaining homeostasis in retina and repair mechanism |
| EI3HA | Eukaryotic translation initiation factor 3 subunit H-A | crucial for protein synthesis, retinal cell maintenance and function |
| TLE3A | Transducin-like enhancer protein 3-A | Involved in neurogenesis, synaptic plasticity and neuronal signaling |
| BI2L2 | Brain-specific angiogenesis inhibitor 1-associated protein 2-like protein 2 | Involved in actin cytoskeleton remodeling |
| SESD1 | SEC14 domain and spectrin repeat-containing protein 1 | Crucial for embryonic neuronal development and signaling |

Table S3: List of Hcfc1 interacting proteins in zebrafish regenerating retina at 2 dpi

| **Protein entry** | **Protein name** | **Function** |
| --- | --- | --- |
| CCM2 | Cerebral cavernous malformations protein 2 homolog | involved in endothelial cell integrity and vascular remodeling, crucial for regenerating retinal vasculature |
| SH3R1 | E3 ubiquitin-protein ligase SH3RF1 | Regulates protein degradation and signaling pathways associated with neuronal survival and differentiation |
| CASPB | Caspase b | Plays a role in apoptotic clearance of damaged cells, facilitating regenerative neurogenesis |
| GATA | Glutamyl-tRNA(Gln) amidotransferase subunit A, mitochondrial | Essential for protein translation and mitochondrial function, supporting metabolic demands during regeneration |
| DDX46 | Probable ATP-dependent RNA helicase DDX46 | Regulates RNA processing and splicing, ensuring proper gene expression during cell proliferation |
| DNM1L | Dynamin-1-like protein | Mediates mitochondrial fission, crucial for cellular energy homeostasis and neuroprotection |
| SIAH2 | E3 ubiquitin-protein ligase Siah2 | Modulates hypoxia response and stress adaptation in regenerating neurons |
| CLU | Clustered mitochondria protein homolog | Associated with stress response and cell survival pathways in neuronal cells |
| JARD2 | Protein Jumonji | Epigenetic regulator controlling chromatin remodeling for neurogenic gene expression |
| STBPA | STAM-binding protein-like A | involved in signal transduction and receptor trafficking during neural development and maintaining homeostasis |
| CTNB1 | Catenin beta-1 | A key player in Wnt signaling, essential for stem cell activation and differentiation |
| CAIAP | CARD- and ANK-domain containing inflammasome adapter protein | Involved in immune response and inflammation resolution, aiding in regenerative microenvironment |
| CCNK | Cyclin-K | Regulates cell cycle progression, ensuring controlled proliferation of retinal progenitor cells |
| NEPRO | Nucleolus and neural progenitor protein | Promotes neural progenitor cell maintenance and differentiation during regeneration |
| KRIT1/CCM1 | Krev interaction trapped protein 1 | Supports vascular integrity and angiogenesis in the regenerating retina |
| TBX5A | T-box transcription factor TBX5-A | Drives retinal cell fate determination and differentiation |
| KLH36 | Kelch-like protein 36 | Involved in cytoskeletal remodeling, facilitating structural reorganization during tissue repair |
| SC4AB | Sodium channel protein type 4 subunit alpha B | Regulates ionic homeostasis crucial for neuronal excitability and functional recovery |
| ATP23 | Mitochondrial inner membrane protease ATP23 homolog | Essential for mitochondrial function, providing energy for regenerative process |

**Uncropped blots**


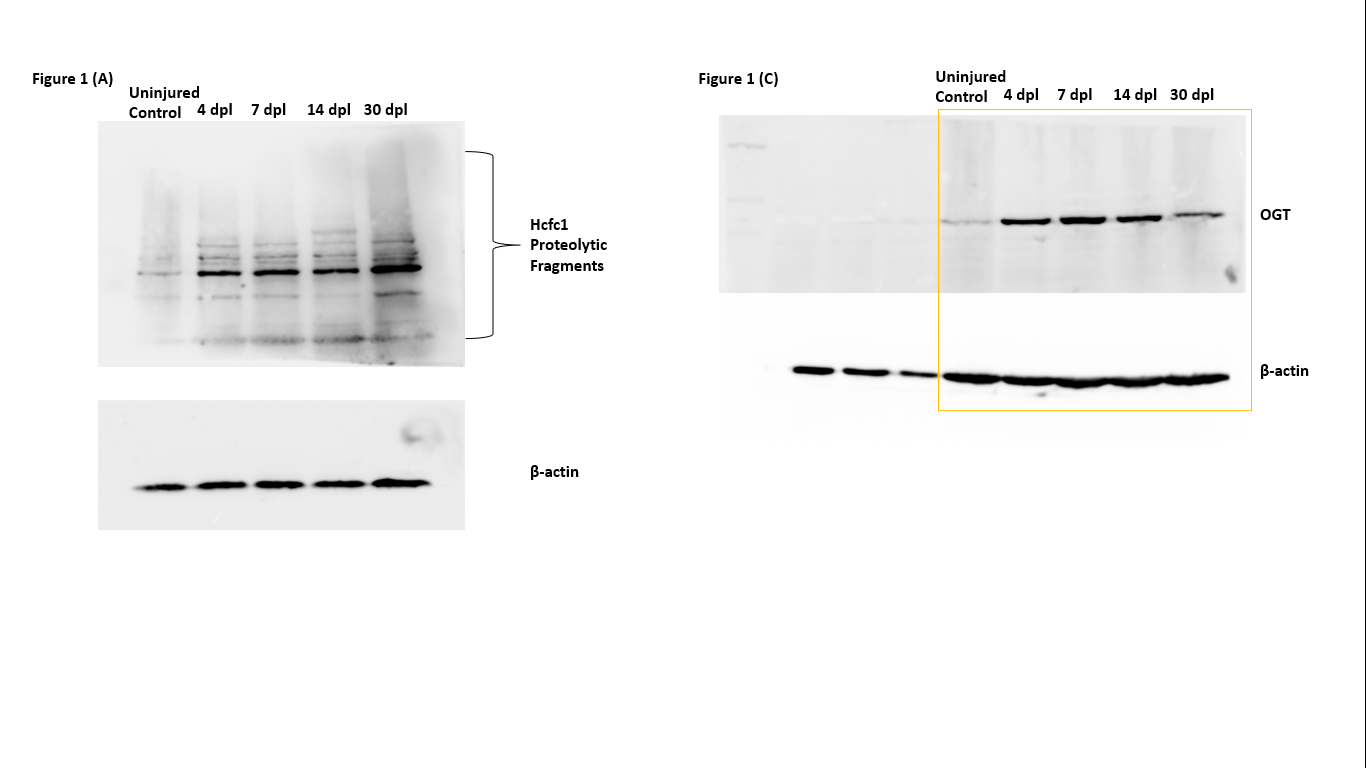


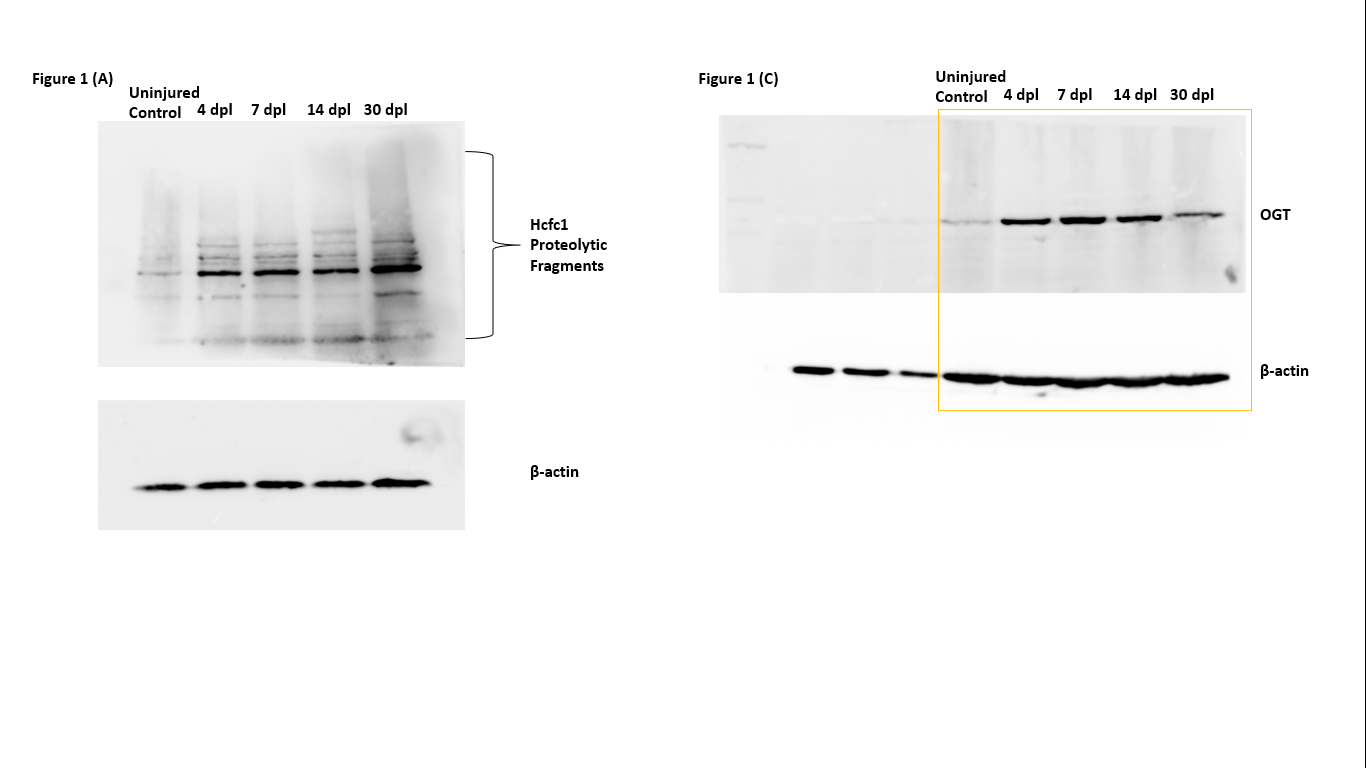


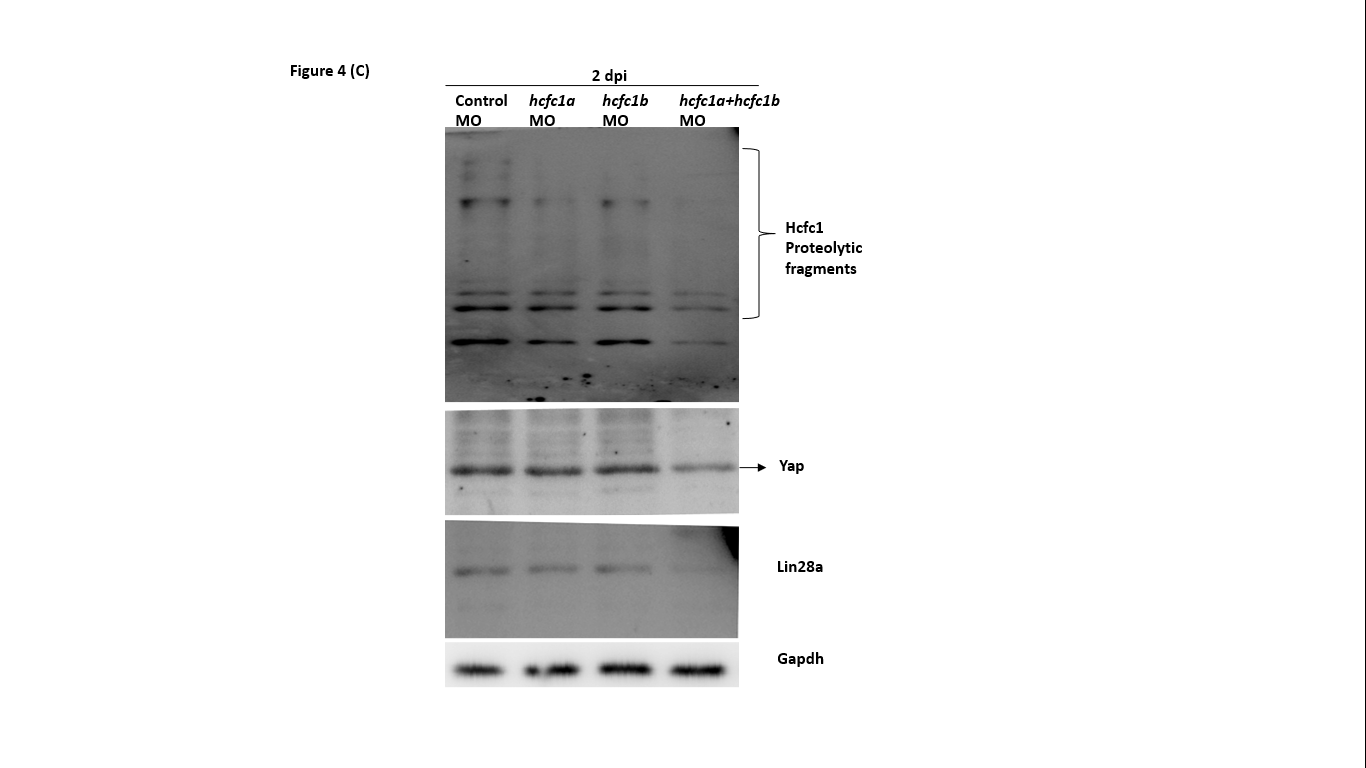


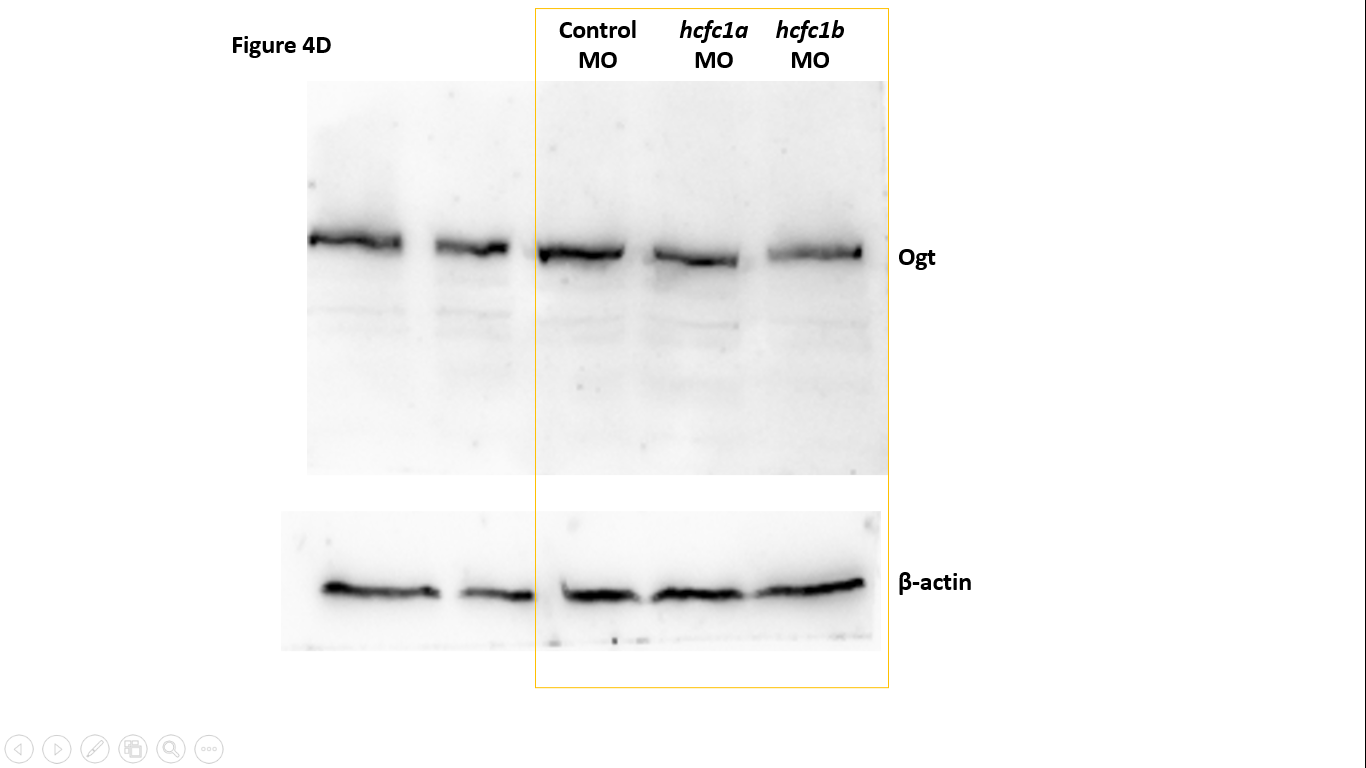


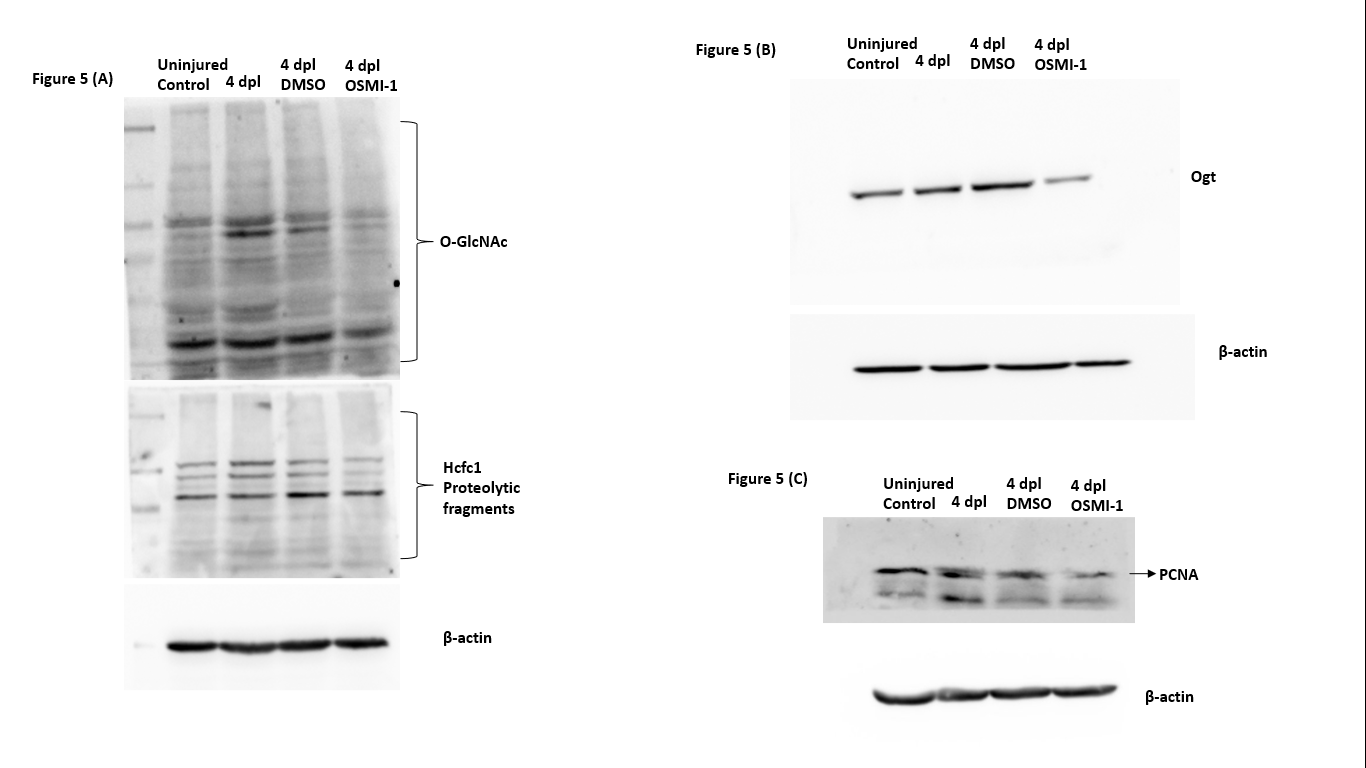


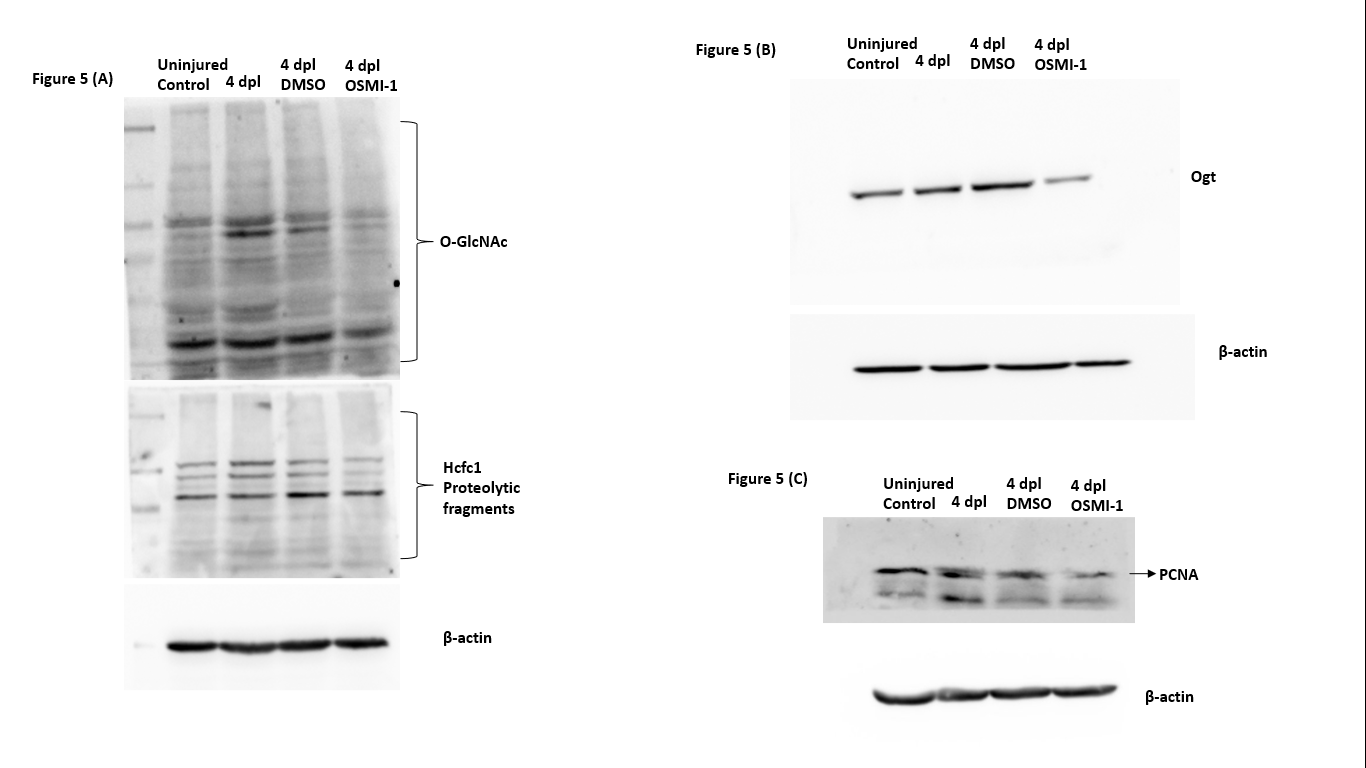


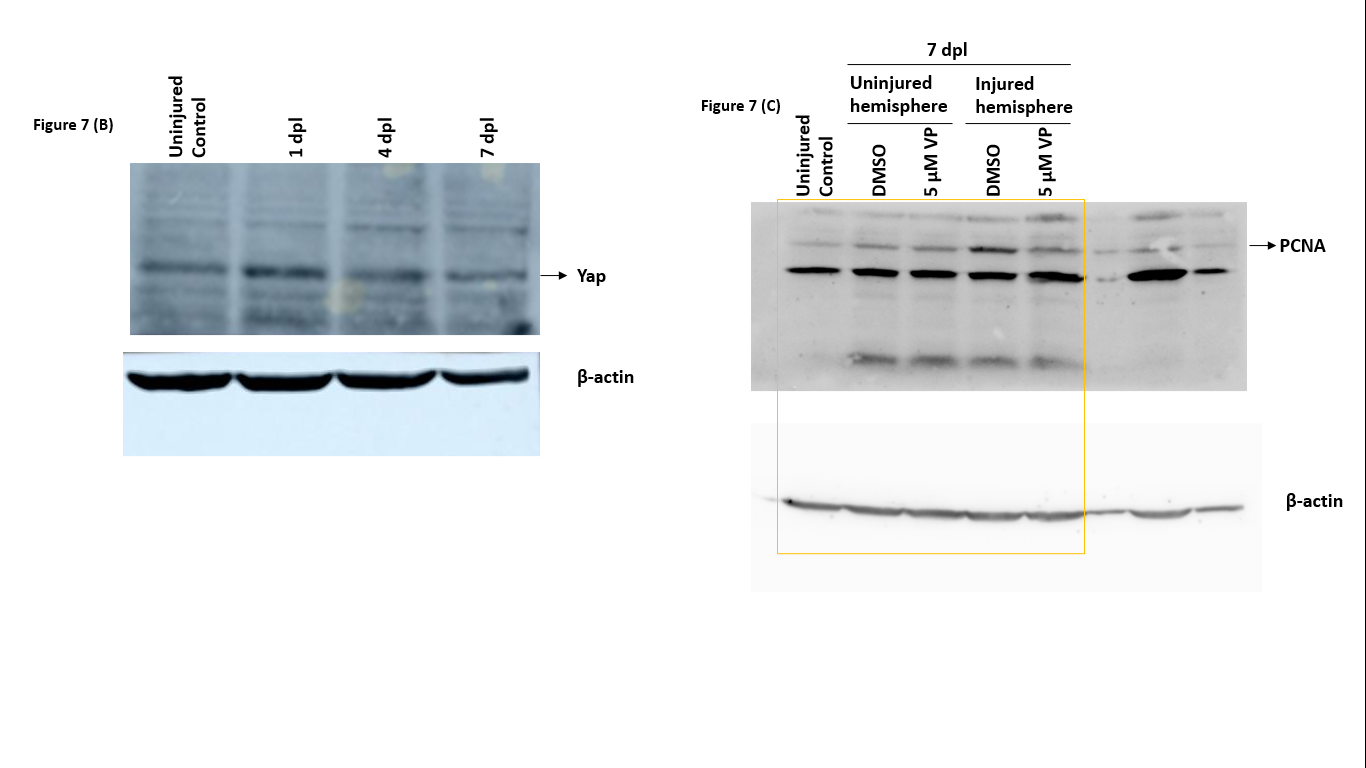


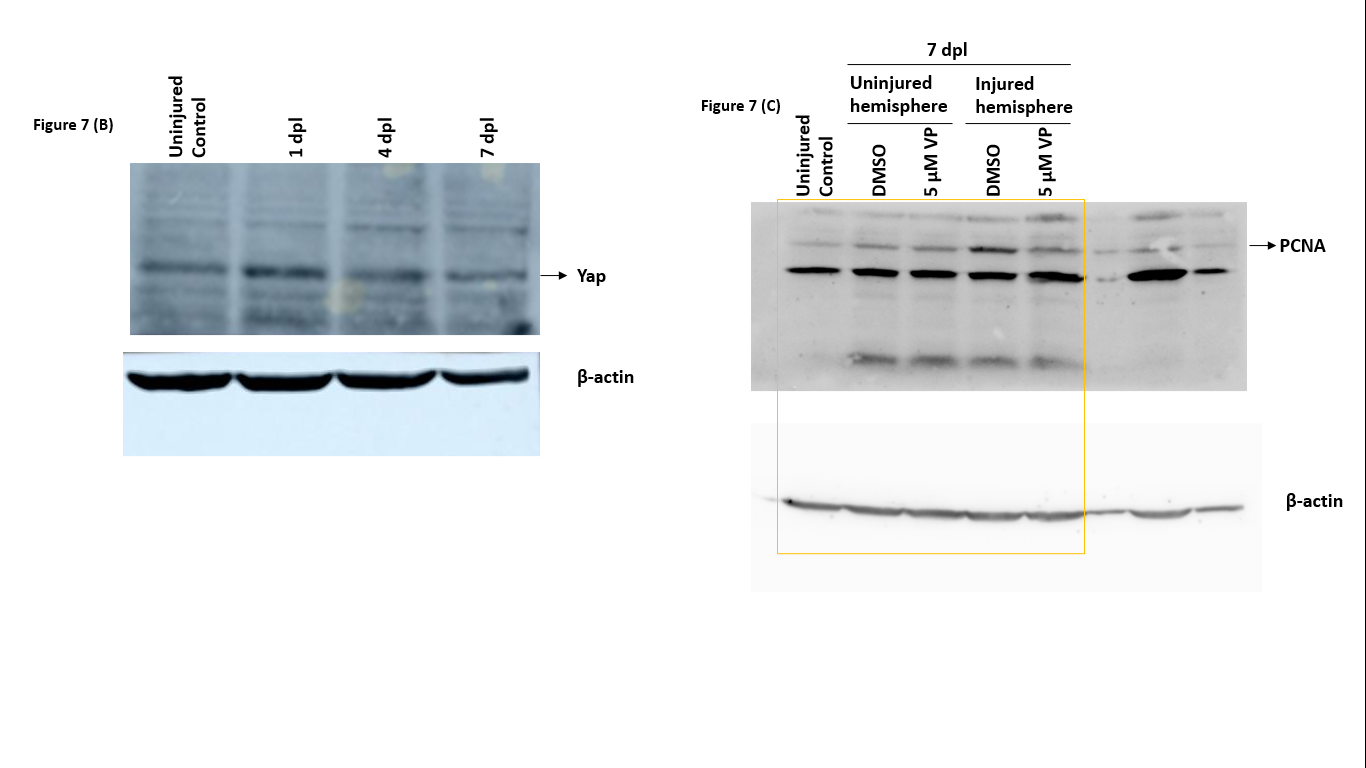


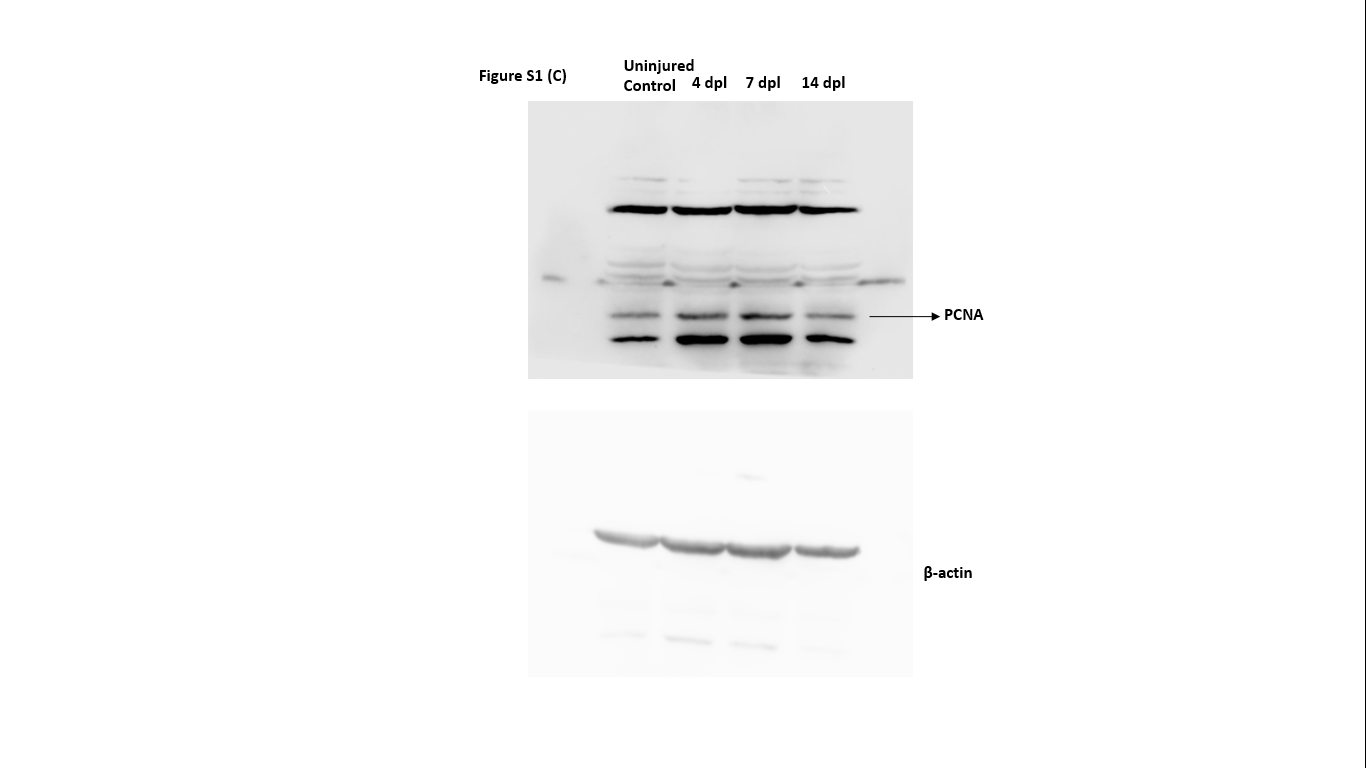


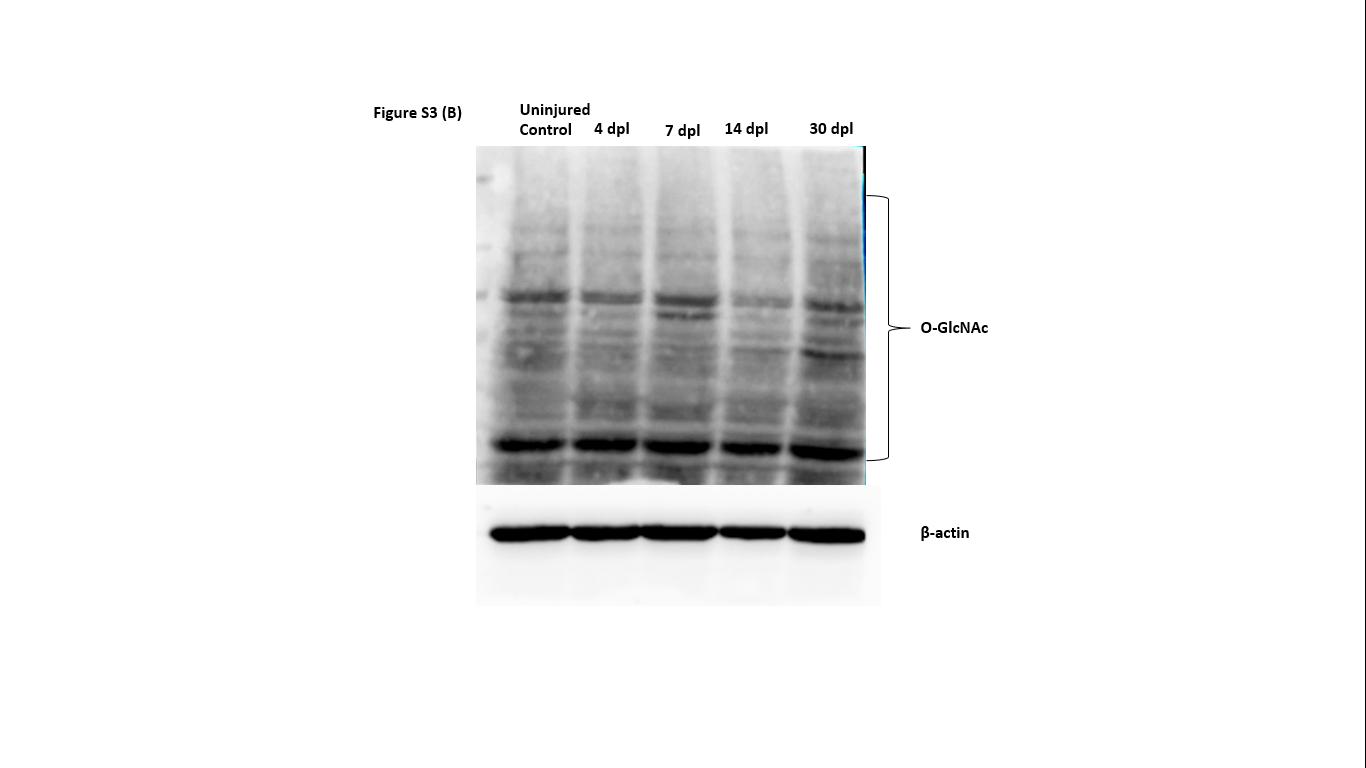


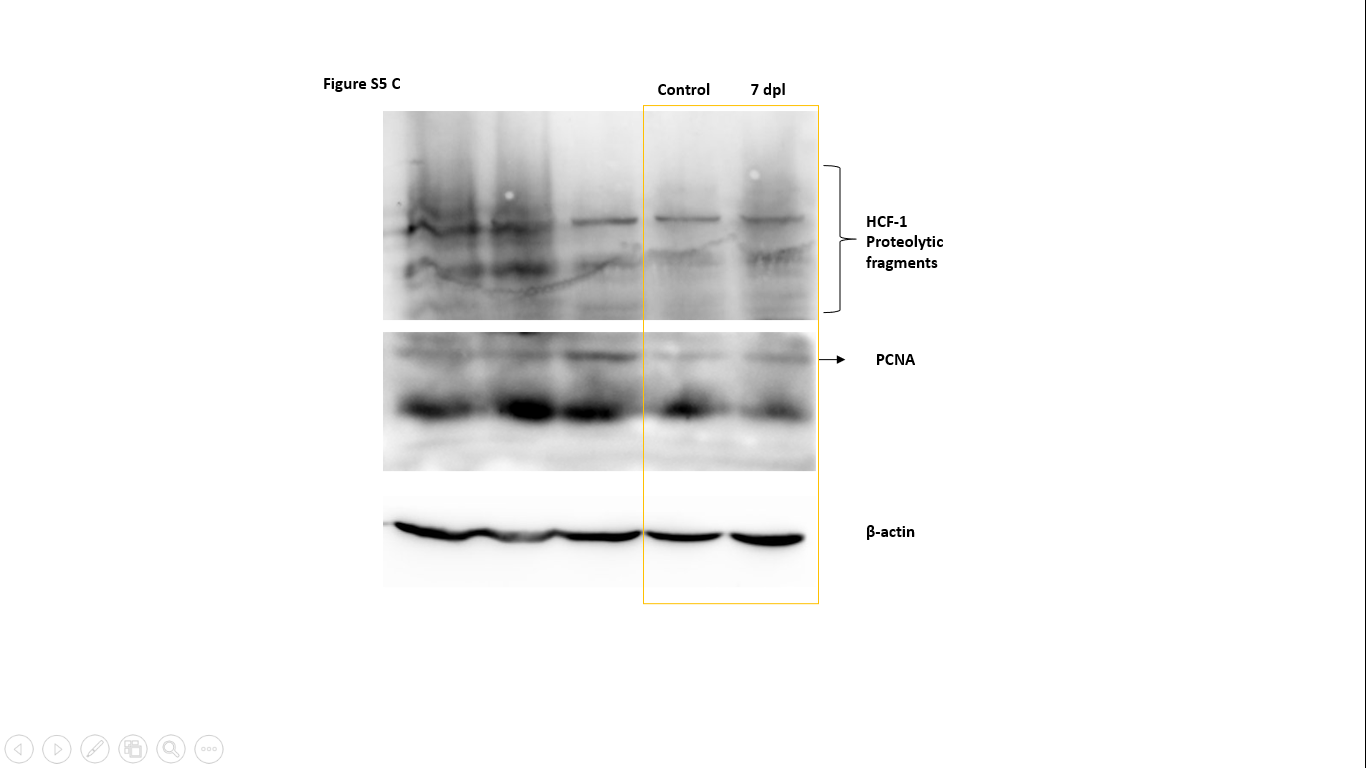


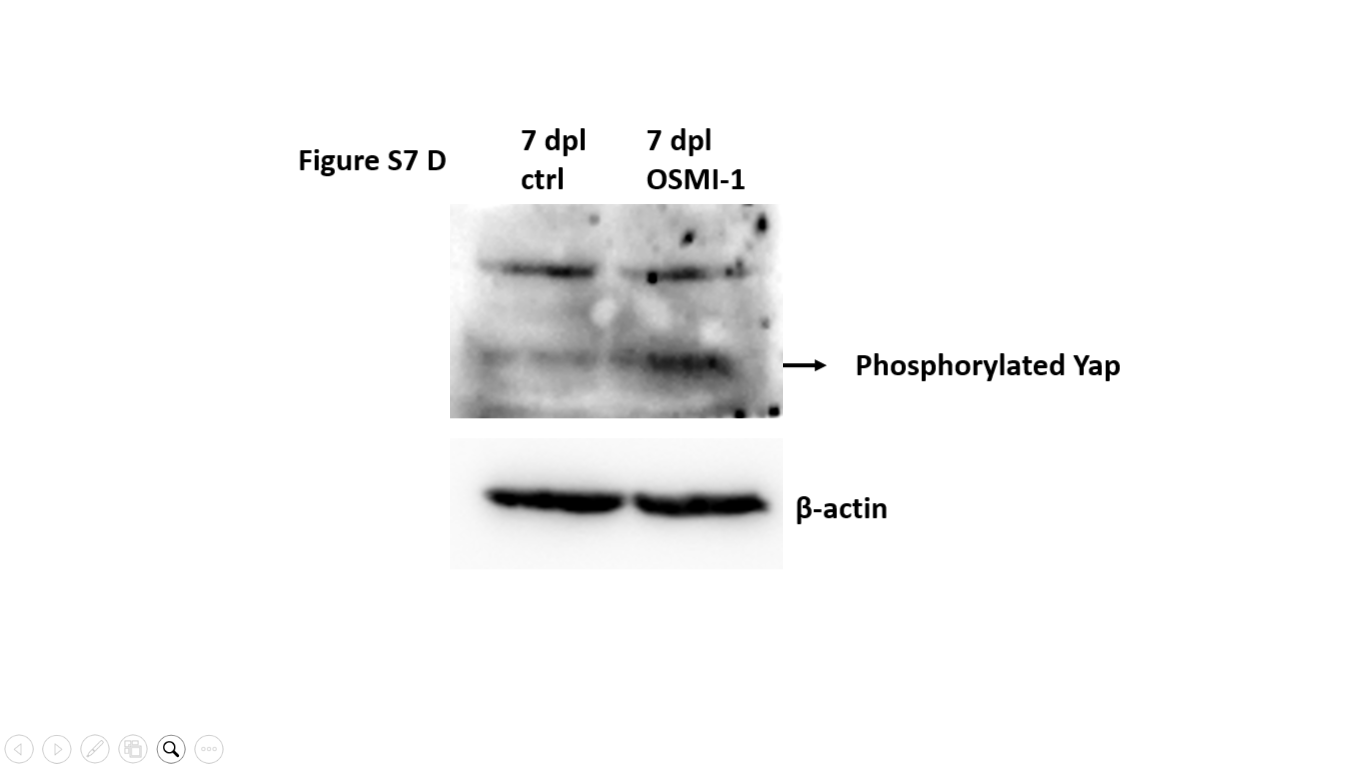


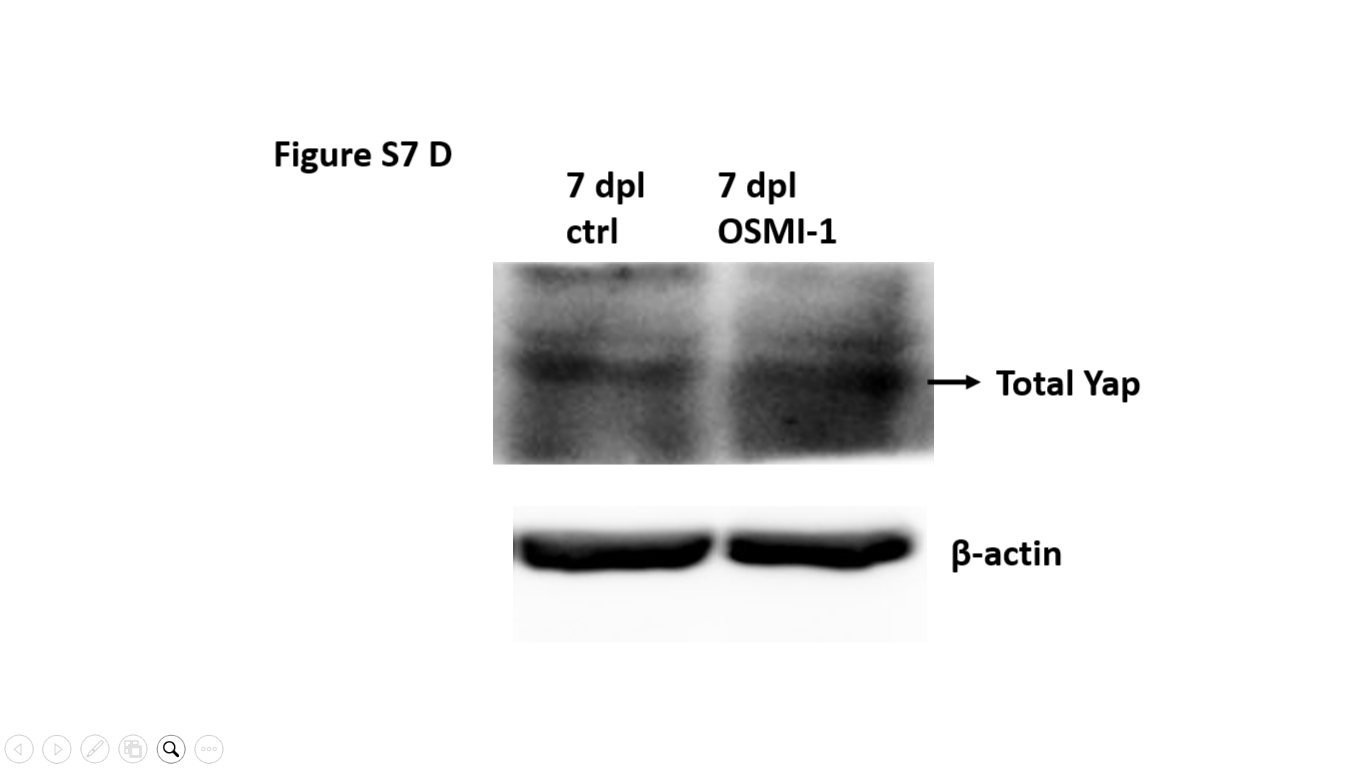


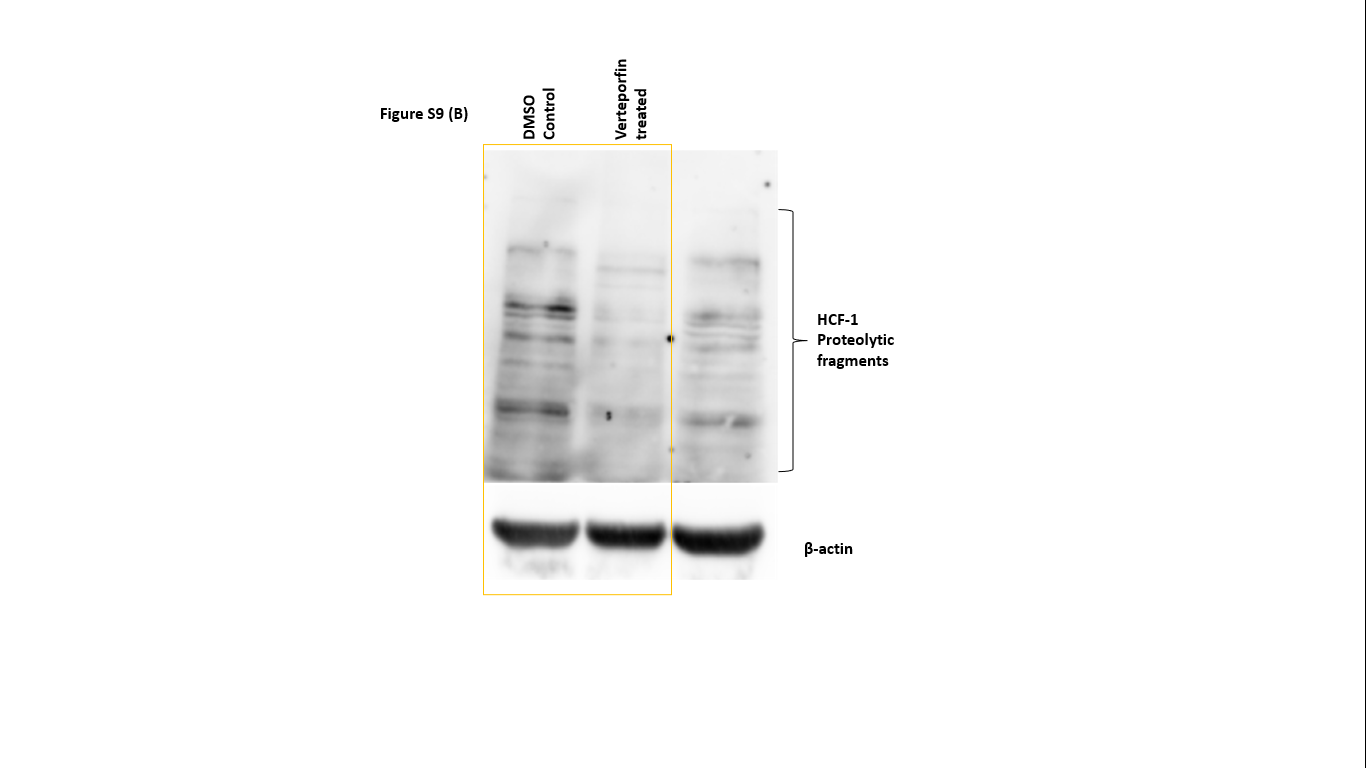

Supplement: Supplementary file 1 — Figure S1: Validation of telencephalic stab‐wound injury model: (A) Telencephalic stab wound‐injury model in Zebrafish brain wherein the right hemisphere is injured with a 30G needle and the left uninjured hemisphere acts as contralateral control. (B) Histological analysis of regenerating brains (1, 4, 7 and 30 dpl) using Toluidine Blue O stain. Scale bar is 100 μm. (C) Immunoblot of uninjured control and regenerating brains with anti‐PCNA (proliferation marker) and anti‐β‐actin as a loading control. (D) Immunostaining of regenerating 4 dpl brain with anti‐BrdU (red) showing co‐localization with DAPI (blue). Scale bar is 100 μm with higher magnification (scale bar 50 μm) in the lower right corner of the merged panel. OB‐ Olfactory bulb, Tel‐ Telencephalon, TeO‐Optic tectum, CC‐Cerebellum. Figure S2: Hcfc1 expression in Zebrafish brain: (A) mRNA expression levels of hcfc1a and hcfc1b in zebrafish brains (n = 3). Significance is represented as n.s. for non‐significant, * for p‐value < 0.05, ** for p‐value < 0.01 and *** for p‐value < 0.001. (B) Immunostaining of control Zebrafish brain telencephalon with anti‐HCFC1 (green). Counterstaining is done with DAPI (blue). Scale bar is 100 μm. Figure S3: O‐GlcNAc and OGT expression during zebrafish brain regeneration: (A) Immunostaining of regenerating brains at 7 dpl with anti‐HCFC1(green) and anti‐OGT (red). Merged panel (A3 and A6) show co‐localization of OGT expressing cells with HCFC1 expressing cells. Scale bar for panels A1‐A3 is 100 μm and for panels A4‐A6 is 50 μm. (B) Immunoblot of regenerating brains (uninjured control, 4, 7, 14 and 30 dpl) with anti‐RL2 (O‐GlcNAc). Anti‐β‐actin was used as a loading control. (C) Quantification of O‐GlcNAc levels during regeneration is shown as a bar graph (n = 3) Significance is represented as n.s. for p‐value > 0.05 and * for p‐value < 0.05. Figure S4: Hcfc1 is upregulated in radial glial progenitor cells during brain regeneration. (A) Immunostaining of zebrafish telencephalon at [file CPR-59-e70132-s001.docx]
